# 5-Fluorouracil: A Narrative Review on the Role of Regulatory Mechanisms in Driving Resistance to This Chemotherapeutic Agent

**DOI:** 10.3389/fonc.2021.658636

**Published:** 2021-04-19

**Authors:** Soudeh Ghafouri-Fard, Atefe Abak, Farhad Tondro Anamag, Hamed Shoorei, Faranak Fattahi, Seyed Alireza Javadinia, Abbas Basiri, Mohammad Taheri

**Affiliations:** ^1^ Dental Research Center, Research Institute for Dental Sciences, Dental School, Shahid Beheshti University of Medical Sciences, Tehran, Iran; ^2^ Department of Medical Genetics, Faculty of Medicine, Tabriz University of Medical Sciences, Tabriz, Iran; ^3^ Faculty of Medicine, Tabriz University of Medical Sciences, Tabriz, Iran; ^4^ Department of Anatomical Sciences, Faculty of Medicine, Birjand University of Medical Sciences, Birjand, Iran; ^5^ Eli and Edythe Broad Center of Regeneration Medicine and Stem Cell Research, University of California, San Francisco, San Francisco, CA, United States; ^6^ Department of Biochemistry and Biophysics, University of California, San Francisco, San Francisco, CA, United States; ^7^ Cellular and Molecular Research Center, Sabzevar University of Medical Sciences, Sabzevar, Iran; ^8^ Urology and Nephrology Research Center, Shahid Beheshti University of Medical Sciences, Tehran, Iran

**Keywords:** lncRNA, miRNA, fluorouracil, expression, biomarker

## Abstract

5-fluorouracil (5-FU) is among the mostly administrated chemotherapeutic agents for a wide variety of neoplasms. Non-coding RNAs have a central impact on the determination of the response of patients to 5-FU. These transcripts via modulation of cancer-related pathways, cell apoptosis, autophagy, epithelial–mesenchymal transition, and other aspects of cell behavior can affect cell response to 5-FU. Modulation of expression levels of microRNAs or long non-coding RNAs may be a suitable approach to sensitize tumor cells to 5-FU treatment via modulating multiple biological signaling pathways such as Hippo/YAP, Wnt/β-catenin, Hedgehog, NF-kB, and Notch cascades. Moreover, there is an increasing interest in targeting these transcripts in various kinds of cancers that are treated by 5-FU. In the present article, we provide a review of the function of non-coding transcripts in the modulation of response of neoplastic cells to 5-FU.

## Introduction

5-fluorouracil (5-FU) and its oral prodrugs including S1 and capecitabine (1) are among the main components of most chemotherapeutic regimens whose efficiencies have been established in the treatment of several neoplasms such as head and neck squamous cell carcinoma (SCC) (2), gastrointestinal SCC and adenocarcinoma (ADC) (3, 4), and SCC of the uterine cervix (5). This agent was introduced by Heidelberger et al. during the 1950s (6). Afterward, it has been increasingly used during the last decades and remained as the backbone of most of chemotherapy regimens. 5-FU has also been used in combination with novel cancer therapies especially targeted therapeutics including vascular endothelial growth factor (VEGF) inhibitors [bevacizumab (7), ziv-aflibercept (8), regorafenib (9) and ramucirumab (10)] and anti-epidermal growth factor receptor (EGFR) therapies [cetuximab (11) and panitumumab (12)]. The cytotoxic effect of 5-FU is mainly induced through inhibition of cellular thymidylate synthase (TS) leading to the prevention of DNA replication (13) and also inhibition of RNA synthesis by the integration of its metabolites into RNA (14) after intracellular activation. These mechanisms of action are, however, only applicable when 5-FU is administered as a single agent chemotherapeutic drug. Since 5-FU has been used in combination with other chemotherapeutics, mostly platinum-based drugs and/or taxanes, and also concurrently during radiotherapy, as a radiosensitizer; other mechanisms of action might be involved that are less clear. Fluorodeoxyuridine monophosphate (FdUMP), fluorodeoxyuridine triphosphate (FdUTP), and fluorouridine triphosphate (FUTP) are all the final active metabolites of 5-FU which are produced after the active transportation of 5-FU into cells by the uracil transport system (15, 16) in addition to the passive paracellular and transcellular routes and also passive diffusion (17, 18). Each of these metabolites prevents cell growth in a specific way. FdUMP inhibits TS leading to indirect DNA damage by deoxynucleotide imbalances and raised levels of deoxyuridine triphosphate. However, FdUTP damages DNA directly by misincorporation into it. In other hand, FUTP is incorporated into RNA resulting in substantial damage in this molecule. Finally, 5-FU causes cell death through simultaneous induction of apoptosis and autophagy (19, 20). Xiong et al. (19) showed that treatment of Bax or PUMA deficient human colon cancer cells with 5-FU resulted in reduction of mTOR activity and subsequent up-regulation of autophagy in this cell line resulting in considerable inhibition of cell proliferation. Moreover, Yang et al. (21) have shown that treatment of human gastric cancer cells with 5-FU resulted in inhibition of cell proliferation through autophagic process resulting from 5-FU-related miR-30 suppression and Beclin-1 upregulation. Besides, mTOR activity and miR-30 suppression as potential pathways for 5-FU-related autophagy activation, Cottone et al. (22) showed that 5-FU can stimulate the inflammatory cells which are responsible for High Mobility Group Box 1 (HMGB1) release resulting in the exacerbation of the autophagy activation. In this context, Nyhan et al. (23) used HMGB1 as a marker of non-apoptotic cell death showing the increase of autophagy after the treatment of human oesophageal cancer cell lines with 5-FU in the presence of miR-193b overexpression. In addition to tumoral cells, non-malignant cells are also influenced by 5-FU-related autophagy. Focaccetti et al. (24) showed clear signs of autophagy along with the significant increase in apoptosis in endothelial cells and cardiomyocytes after treatment with 5-FU. Interestingly, more recently autophagy is proposed as a potential way of acquired resistance of tumoral cells towards 5-FU (25). Globally, the alternation of TS coding gene, i.e. TYMS (26), single nucleotide polymorphisms affecting the activity of methylenetetrahydrofolate reductase (MTHFR) (27), and Dihydropyridine dehydrogenase (DPD) expression (28) are considered as the main mechanisms of resistance.

## Catabolic and Anabolic Way of 5-Fluorouracil Transformation

5-FU is a uracil analog with a fluorine atom at the C-5 position. After intravenous administration of 5-FU, this medication can rapidly enter target cells using the same transport mechanism as uracil (15). Accumulating evidence demonstrated that the transportation of 5-FU could be passively triggered via transcellular and paracellular pathways in tumor cell monolayers. In addition, 5-FU by passive diffusion can promptly pass the blood-brain barrier (BBB) (18, 29). 5-FU can be transformed to the following active metabolites in the target cells: 1) Fluorodeoxyuridine triphosphate (FdUTP) that could combine into DNA rather than deoxythymidine triphosphate (dTTP); 2) Fluorouridine triphosphate (FUTP) that could combine into RNA rather than uridine triphosphate (UTP). FUTP alters RNA function and processing, and FdUTP and FdUMP can induce DNA damage. Both of these procedures have a profound effect on RNA and DNA triggering cell death in tumor cells; and 3) FdUMP suppresses the function of Thymidylate synthase (TS) in the ternary complex (16). FdUMP can create a constant ternary complex with 5, 10-methylenetetrahydrofolate (CH2THF), and TS. TS can in turn catalyze conversion of deoxyuridine monophosphate (dUMP) to deoxythymidine monophosphate (dTMP) (1). The ternary complex could impede the availability of dUMP to the nucleotide-binding site (NBS) of TS via competing with FdUMP that could lead to imbalance in deoxynucleotides pool, particularly enhancing deoxyuridine triphosphate (dUTP) levels and causing damage to DNA. Reduction of dTMP could induce reduction of dTTP, which disrupts the levels of the other deoxynucleotides (30). Another 5-FU activation cascade includes thymidine phosphorylase (dThdPase) that could trigger the transformation of 5-FU to fluorodeoxyuridine (FUDR) which could be phosphorylated via thymidine kinase (TK) to FdUMP. Phosphorylation reaction through the UrdPase needs ribose-1-phosphate as a cofactor that could play an effective role in creating FUMP. On the other hand, the phosphorylation reaction via dThdPase needs deoxyribose-1-phosphate as a cofactor resulting in the creation of FdUMP. Subsequently, FUMP could be phosphorylated to fluorouridine diphosphate (FUDP) that is either subsequently phosphorylated to the active metabolite FUTP or could be transformed to fluorodeoxyuridine diphosphate (FdUDP) via ribonucleotide reductase (16). Afterward, FdUDP is either phosphorylated to FdUTP or could be dephosphorylated to FdUMP. Therefore, both dUTP and FdUMP could effectively contribute to DNA damage. The transformation of 5-FU to FdUMP in the alimentary canal and bone marrow could in turn lead to digestive tract toxicity as well as myelotoxicity (1). Additionally, dihydropyrimidine dehydrogenase (DPYD), which is a ubiquitous and rate-limiting enzyme of the uracil catabolic pathway and is present in the liver, gut, intestinal mucosa, and several other tissues, could be considered as a major enzyme for the degradation of 5-FU (1, 31). 5-FU represents poor bioavailability because of its prompt catabolic degradation to 5, 6-dihydro-5-fluorouracil (DHFU) via DPYD (25). DPYD could catabolize 5-FU to 5, 6-dihydro-5-fluorouracil (DHFU), eventually resulting in the creation of α-fluoro-β-ureidopropionic acid (FUPA) as well as α-fluoro-β-alanine (FBAL) that subsequently could be excreted through the kidneys (32). Oral administration of 5-FU in the form of 5-FU pro-drugs (oral FPs) could result in imperfect and disordered bioavailability because of variation in the function of DPYD. Hence, it is correlated to unpredictable levels of 5-FU in the plasma because of noticeable intra- and inter-patient mutability in its adsorption/deletion (33).

## Non-Coding RNAs and Response to 5-FU

Non-coding RNAs modulate several cellular pathways that are involved in the response of neoplastic cells to 5-FU or its oral prodrugs (34). In a broad classification, we categorize non-coding RNAs to long non-coding RNAs (lncRNAs) and microRNAs (miRNAs) with sizes more than 200 nucleotides and about 22 nucleotides, respectively. These two main classes of non-coding RNAs vary in their mode of action in the regulation of gene expression, however, both regulate fundamental aspects of cellular activities among them are apoptosis, autophagy, and DNA repair (35, 36). These cellular processes define the response of neoplastic cells to chemotherapeutic agents such as 5-FU.

## miRNAs and 5-FU Response

The impact of miRNAs in the modulation of 5-FU resistance has been largely assessed in colorectal cancer (CRC) cells. For instance, as a tumor suppressor miRNA, expression of miR-15b-5p has been down-regulated in tissues and cells obtained from patients with this kind of neoplasm. Up-regulation of miR-15b-5p has enhanced 5-FU-associated cell apoptosis and ameliorated cell response to 5-FU both *in vitro* and in animal models. NF-κB signaling pathway has been identified as the mediator of miR-15b-5p effect on response to 5-FU. This miRNA negatively regulates NF-κB1 and IKK-α. miR-15b-5p has also been shown to target the anti-apoptotic gene *XIAP* (37). In CRC cells, miR-21 influences response to 5-FU through targeting PDCD4 and hMSH2 (38, 39). Notably, PDCD4 has also been shown to be targeted by miR-1260b. This miRNA confers resistance to 5-FU and inhibits apoptosis in CRC cells via the PI3K/Akt signaling pathway (40). Expression of miR-21 has been considerably increased in exosomes of CRC cells versus normal human colon epithelium. Exosomal miR-21 can enhance the expression of genes participating in cell proliferation, invasiveness, and extracellular matrix construction. Moreover, miR-21 through targeting PDCD4 can increase resistance to 5-FU (38). Another experiment in this kind of cancer has shown the role of miR-22 in the enhancement of sensitivity to 5-FU through inhibition of autophagy and boosting apoptosis. These effects of miR-22 are mediated through the suppression of expression of B-cell translocation gene 1 (BTG1) (41). Treatment of CRC cells with 5-FU has resulted in enhancement of miR-23a while down-regulation of APAF-1 in these cells. miR-23a antisense has enhanced the activation of the caspases-3 and -7 through up-regulation of miR-231 target APAF-1 and increased the 5-FU-associated apoptosis. Yet, miR-23a antagonism did not surge the anticancer impact of 5-FU in the xenograft model of CRC (42). The tumor-suppressive miRNA miR-199b directly targets the PP2A inhibitor SET, a crucial factor in conferring resistance to 5-FU. An experiment in rectal cancer cells has shown that both miR-199b up-regulation and SET suppression can combat 5-FU resistance. Expression of miR-199b has been decreased in about one-fourth of cases in association with lymph node positivity following chemoradiotherapy and advanced stage (43). **Table 1** summarizes the influence of miRNAs on response of CRC cells to 5-FU.

**Table 1:** Role of miRNAs in the modulation of response to 5-FU in colorectal cancer (ANT, adjacent normal tissue).

microRNA	Animal/Human	Cell line	Targets/Regulators	Function	Ref
miR-15	62 pairs of CRC and ANTs	HCT116	Bcl-2, Bcl-XL, NF-kB	MiR-15 could sensitize CRC cells to 5-FU and increase apoptosis *via* NF−κB.	(37)
miR-21	–	HT29, T84, LS174, CRL1831	PDCD4, TPM1, PTEN	MiR-21 by targeting PDCD4 could promote proliferation, invasion and therapy resistance.	(38)
miR-21	–	HT-29, HT-29/5-FU	hMSH2, TP, DPD	MiR-21 by targeting hMSH2 could increase cell proliferation and chemoresistance and inhibit apoptosis.	(39)
miR-21	mouse	Colo-320DM, SW620, HCT-116, SW480, RKO	hMSH2	MiR-21 by targeting hMSH2 could induce resistance to 5-FU in CRC cells.	(44)
miR-22	mouse/human; 94 pairs of CRC and ANTs	SW620, RKO	BTG1	MiR-22 by targeting BTG1 could increase 5-FU sensitivity via inhibiting autophagy and promoting apoptosis in CRC cells.	(41)
miR-23a	mouse	HCT116, HT29	APAF-1, Caspase-9	MiR‐23a by targeting the APAF‐1/Caspase‐9 axis could enhance 5‐FU resistance in CRC cells.	(42)
miR-24	–	HCT116, RKO, SW480, SW48, CCD-18Co	DND1	MiR-24 by targeting DND1 could enhance apoptosis and sensitivity in CRC cells.	(45)
miR-26b	mouse/human; 36 CRC tissues and 16 normal ANTs	HT-29, LOVO, HT-29/5-FU, LOVO/5-FU, FHC	Pgp	MiR-26b by targeting Pgp could enhance chemosensitivity to 5-FU.	(46)
miR-29c-3p	–	HCT116 p53+/+, HCT116 p53−/−	PHLDB2	MiR-29c-3p by targeting PHLDB2 could suppress colon cancer cell invasion and migration.	(47)
miR-30-5p	30 pairs of CRC and ANTs	Caco2, HT29, HCT15, HCT116, SW620, SW480, 293T	USP22, Wnt/β-catenin	MiR-30-5p by targeting USP22 could suppress cell chemoresistance and stemness in CRC cells through the Wnt/β‐catenin signaling pathway.	(48)
miR-31	mouse/human; 112 pairs of CRC and ANTs	DLD‐1, SW480, WiDr, HT‐29, SW48, DLD/F, SW/F	FIH-1	MiR-31 by silencing FIH-1 could contribute to CRC cell resistance to 5-FU.	(49)
miR-34a	–	DLD-1, DLD-1/5FU	Sirt1, E2F3, PI3K/Akt	MiR-34a targets Sirt1 and E2F3 genes and decreases resistance to 5-FU.	(50)
miR-34a	mouse	SW480, LoVo	DLL1, Notch	MiR-34a by targeting DLL1 could overcome ABCG2-mediated resistance to 5-FU in CRC cells via the Notch signaling pathway.	(51)
miR-122	mouse	HCT-116/R, HT-9/R, HCT-116, HT-29	PKM2	MiR-122 by inhibiting PKM2 could reverse chemoresistance for 5-FU in CRC cells.	(52)
miR-129	mouse/human: 77 pairs of CRC and ANTs	HCT116, RKO, SW480	Bcl-2, E2F3, TS	MiR-129 by targeting Bcl-2 could promote apoptosis, inhibit cell proliferation, cause cell-cycle arrest, and also increase response to 5-FU in CRC cells.	(53)
miR-133b	–	HT29, HCT116, SW620, 293T	DOT1L	MiR-133b by targeting DOT1L could suppress CRC cell stemness and chemoresistance.	(54)
miR-135b/-182	mouse/human; 31 pairs of CRC and ANTs	HCT-8, LoVo, HCT-8/5-FU, LoVo/5-FU	ST6GALNAC2, PI3K/Akt	MiR-135b and miR-82 by targeting ST6GALNAC2 could promote chemoresistance of CRC cells via the PI3K/Akt signaling pathway.	(55)
miR-139-5p	mouse/human; 204 CRC tissues and 54 normal healthy controls	HT29, LS174T, SW480, SW620, RKO, HCT116, COLO205, LoVo, NCM460	Bcl-2, EMT	MiR-139-5p by targeting Bcl-2 could sensitize CRC cells to 5-FU via EMT regulation.	(56)
miR-139-5p	–	HCT-116, LoVo, HCT-8, HCT-116/5-FU, HCT-8/5-FU	NOTCH-1	MiR-139-5p by targeting NOTCH-1 could sensitize CRC cells to 5-FU.	(57)
miR-143	–	HCT116, SW480, LoVo, SW620	ERK5, Bcl-2, NF-kB	MiR-143 by reducing NF-kB, ERK5 and Bcl-2 could increase 5-FU cytotoxicity in CRC cells.	(58)
miR-145	mouse/human; 152 pairs of CRC and ANTs	SW620, 5-FuR SW620	RAD18	MiR-145 by directly targeting DNA damage-related gene RAD18 could reverse drug resistance in CRC cells.	(59)
miR-149	24 CRC tissues	HCT-8, LoVo, HCT-8/5-FU, LoVo/5-FU	FOXM1	MiR-149 by targeting FOXM1 could increase sensitivity to 5-FU in CRC cells.	(60)
miR-185-3p	120 pairs of CRC and ANTs	HCT-116, HCT-8, HCT-116/5-FU, HCT-8/5-FU	AQP5, EMT	MiR-185-3p by targeting AQP5 could enhance chemosensitivity in CRC cells via EMT regulation.	(61)
miR-195	–	HCT-116	WEE1, CHK1	MiR-195 by targeting WEE1 and CHK1 could regulate the cell cycle and desensitize CRC cells to 5-FU.	(62)
miR-195-5p	15 pairs of CRC and ANTs	Caco-2, HCT8, HCT116, SW480	GDPD5	MiR-195-5p by targeting GDPD5 could inhibit metastasis and sensitize CRC cells to 5-FU.	(63)
miR-200c	–	HCT-116	E-cadherin, PTEN	Inhibition of miR-200c could trigger the acquired resistance of CRC cells to 5-FU.	(64)
miR-203	mouse	FHC, HCT-116, Caco2, SW480, LoVo/5-FU	TYMS	MiR-203 by targeting TYMS could enhance chemosensitivity to 5-FU in CRC cells.	(65)
miR-204	33 pairs of CRC and ANTs	LoVo, HT29, SW620, SW116, HCT116, SW480, HcoEpiC	HMGA2	MiR-204 by inhibiting HMGA2 could enhance sensitivity to 5-FU.	(66)
miR-206	mouse	HCT116, RKO, HCT116/FR, RKO/FR	Bcl-2	MiR-206 by targeting Bcl-2 could decrease 5-FU resistance in colon cancer cells.	(67)
miR-210-3p	–	HT29, HT29R	SDHD, RAD-52	MiR-210-3p by targeting RAD-52 could increase DNA damage repair and by targeting SDHD and could induce a shift from oxidative metabolism towards OXPHOS.	(68)
miR-214	–	HT-29, LoVo, HT-29/5-FU, LoVo/5-FU	Hsp27	MiR-214 by targeting Hsp27 could sensitize CRC cells to 5-FU.	(69)
miR-215-3p	mouse/human; 56 CRC tissues and 23 normal tissues	HCT116/5-FU, HCT116, LoVo, HT-29, SW480	CXCR1	MiR-215-3p by targeting CXCR1 could improve the 5-Fu sensibility in the colorectal cancer cell.	(70)
miR-302a	24 pairs of CRC and ANTs	HCT116, HT29	IGF-1R, AKT	MiR-302a by targeting IGF-1R increases 5-FU-induced cell death in CRC cells.	(71)
miR-329	56 pairs of CRC and ANTs	HCT116, SW480	E2F1	MiR-329 by targeting E2F1 could inhibit viability, and invasion and also enhance sensitivity to 5-FU in CRC cells.	(72)
miR-330	59 pairs of CRC and ANTs	HCT116, HT29, SW480, SW620, FHC, 293T	TYMS	MiR-330 by targeting TYMS could inhibit cell proliferation and enhance chemosensitivity to 5-FU in CRC cells.	(73)
miR-361	–	HCT116, HT29, HCT116−Res, HT29−Res	FOXM1, ABCC5/10	MiR-361 by targeting FOXM1-ABCC5/10 could sensitize resistant CRC cells to 5-FU, inhibit colony formation, and induce apoptosis.	(74)
miR-375-3p	mouse, TCGA dataset	HCT116, HT29, SW480, Caco2, NCM460, HCT-15/FU	TYMS	MiR-375-3p by targeting TYMS could increase 5-FU sensitivity by enhancing cell apoptosis and cell cycle arrest and suppression of cell proliferation, migration, and invasiveness.	(75)
miR-425-5p	mouse	HCT116-R, HCT116	PDCD10	MiR-425-5p by targeting PDCD10 could increase resistance to 5-FU in CRC cells.	(76)
miR-488	280 pairs of CRC and ANTs	SW620, HT-29, Lovo, HCT116, NCM-460	PFKFB3, glycolysis	MiR-488 by targeting PFKFB3 could alleviate chemoresistance for 5-FU and glycolysis of CRC cells.	(77)
miR-494	mouse	HCT116, HCT15, HCT8, HT-29, LoVo, SW480/5-Fu	DPYD	MiR-494 by targeting DPYD could enhance apoptosis and increase chemosensitivity to 5-FU.	(78)
miR-519d	–	HCT116, SW480	CCND1	MiR-519d by targeting CCND1 could reverse resistance to 5-FU in CRC cells.	(79)
miR-543	–	HCT8, HCT8/FU	PTEN, PI3K/Akt	MiR-543 by targeting PTEN could promote cell migration, inhibit apoptosis, and induce chemoresistance to 5-FU.	(80)
miR-552	mouse/human: 97 pairs of CRC and ANTs	SW-480, SW-620, HT-116, CCD-18Co	SMAD2, TGF-β	MiR-552 by targeting SMAD2 could enhance 5-FU sensitivity in CRC cells via TGF−β signaling pathway.	(81)
miR-577	mouse/human; 64 pairs of CRC and ANTs	SW480, SW620, CaCo2, HT29, Lovo, HCT-116, NCM460	HSP27	MiR-577 by targeting HSP27 could suppress tumor growth and enhance chemosensitivity in CRC cells.	(82)
miR-587	mouse/human	RKO, HCT116, FET, GEO	PPP2R1B, AKT	MiR-587 by targeting PPP2R1B could antagonize 5-FU-induced apoptosis and confer chemoresistance.	(83)
miR-874	mouse/human; 32 pairs of CRC and ANTs	LoVo, SW1116, SW480, HCT-116, NCM460	XIAP	MiR-874 by targeting XIAP could inhibit growth, induce apoptosis, and reverse chemoresistance in CRC cells.	(84)
miR-1260b	30 pairs of CRC and ANTs	HCT116, SW480	PDCD4, IGF1, PI3K/Akt	MiR-1260b by targeting PDCD4 could confer resistance to 5-FU and inhibit apoptosis in CRC cells via the PI3K/Akt signaling pathway.	(85)
miR-199b	110 pairs of locally advanced rectal cancer and ANTs	SW480, HT-29, SW480R	SET	MiR-199b downregulation by targeting SET could confer resistance to 5-FU in locally advanced rectal cancer cells.	(43)

Hepatocellular carcinoma (HCC) is another type of cancer in which the role of miRNAs in the regulation of response to 5-FU has been vastly assessed. Forced over-expression of miR-122 in hepatoblastoma cells has reduced expressions of Bcl-2 and Bcl-XL while increasing P53 protein levels. These effects have been accompanied by enhancement of apoptosis and higher sensitivity to 5-FU, demonstrating the impact of this miRNA in response to 5-FU (86). Another experiment in several HCC cell liens has shown lower expression of miR-125b in 5-FU-resistant cells compared with sensitive cells. TRansfection of pre-miR-125b into HCC cells has led to the improvement of sensitivity to 5-FU. F-FU resistant cells also exhibited higher glucose uptake and lactate synthesis compared with 5-FU-sensitive cells. Remarkably, miR-125 has been shown to decrease glucose metabolism by influencing the expression of hexokinase II (87). miR-147 is a tumor suppressor miRNA whose expression has been reduced in HCC cell lines and clinical samples. Overexpression of miR-147 has suppressed *in vitro* proliferation and migration of HCC cells and enhanced cytotoxic effects of 5-FU. Moreover, it has decreased *in vivo* tumorigenicity of HCC cells. HOXC6 has been recognized as the downstream target of miR-147 through which this miRNA enhances 5-FU sensitivity (88). Another tumor suppressor miRNA in HCC namely miR-503 regulates the expression of EIF4E and enhances response to 5-FU (89). **Table 2** demonstrates the impact of miRNAs modulation of response to 5-FU in HCC cells.

**Table 2:** Role of miRNAs in the modulation of response to 5-FU in hepatocellular carcinoma (ANT, adjacent normal tissue).

microRNA	Animal/Human	Cell line	Targets/Regulators	Function	Ref
miR-122	–	BEL-7402, BEL-7402/5-FU	Bcl-2, Bcl-XL, p53	MiR-122 by downregulating Bcl-2 and Bcl-XL and increasing p53 could enhance HCC cells sensitivity to 5-FU and induce cell death.	(86)
miR-125b	–	SMMC-7221, Huh7, MHCC-97L, HepG2, HepG3, BEL-7402, THLE-2, THLE-3	HK II, glycolysis	MiR-125b by targeting HK II could sensitize HCC cells to 5-fluorouracil through inhibition of glycolysis.	(87)
miR-133a/-326	–	HepG2	Bcl−XL	MiR-133a/-326 by directly targeting Bcl-XL could co-contribute to HCC cell 5-FU sensitivity.	(90)
miR-141	–	HepG2, HuH7,SMMC-7721, HepG2/5-FU, SMMC-7721/5-FU, HuH7/5-FU	Keap1, Nrf2	MiR-141 by repressing Keap1 could confer 5-FU resistance and contribute to reduced susceptibility to 5-FU-induced apoptosis via activating Nrf2-dependent antioxidant pathway.	(91)
miR-145	mouse/human: 102 pairs of HCC and ANTs	SNU449, Huh7, LO2	TLR4	MiR-145 by targeting TLR4 could enhance chemosensitivity in HCC cells.	(92)
miR-147	mouse/human; 10 pairs of HCC and ANTs	HepG2, C3A, SNU-398, Hep3B, THLE2, THLE3, HiH7, MHCC97L, MHCC97H	HOXC6	MiR-147 by inhibiting HOXC6 could suppress HCC cell proliferation, migration and enhance chemosensitivity to 5-FU.	(88)
miR-193a-3p	mouse	QGY-7703, SMMC-7721, BEL-7402, HepG2, Hep3B, PLC, YY-8103, FOCUS	SRSF2, E2F1	DNA methylation-regulated miR-193a-3p by repressing SRSF2 could dictate resistance of HCC cells to 5-FU.	(93)
miR-195	–	BEL-7402, BEL-7402/5-FU	Bcl-w	MiR-195 by targeting Bcl-w could confer HCC cells to 5-FU-induced apoptosis.	(94)
miR-200a-3p	–	Hep3B	DUSP6	MiR-200a-3p by regulating DUSP6 expression could increase 5-FU resistance in Hep3B cells.	(95)
miR-302b	–	SMMC-7721, HepG2	Mcl-1, DPYD	MiR-302b by targeting Mcl-1 and DPYD could enhance the sensitivity of HCC cells to 5-FU.	(96)
miR-503	9 HCC and ANTs	HepG2, BEL-7402, SMMC-7721, L-02	EIF4E	MiR-503 by targeting EIF4E could render HCC cells susceptible to 5-FU and inhibit cell proliferation.	(89)

**Figure 1:**
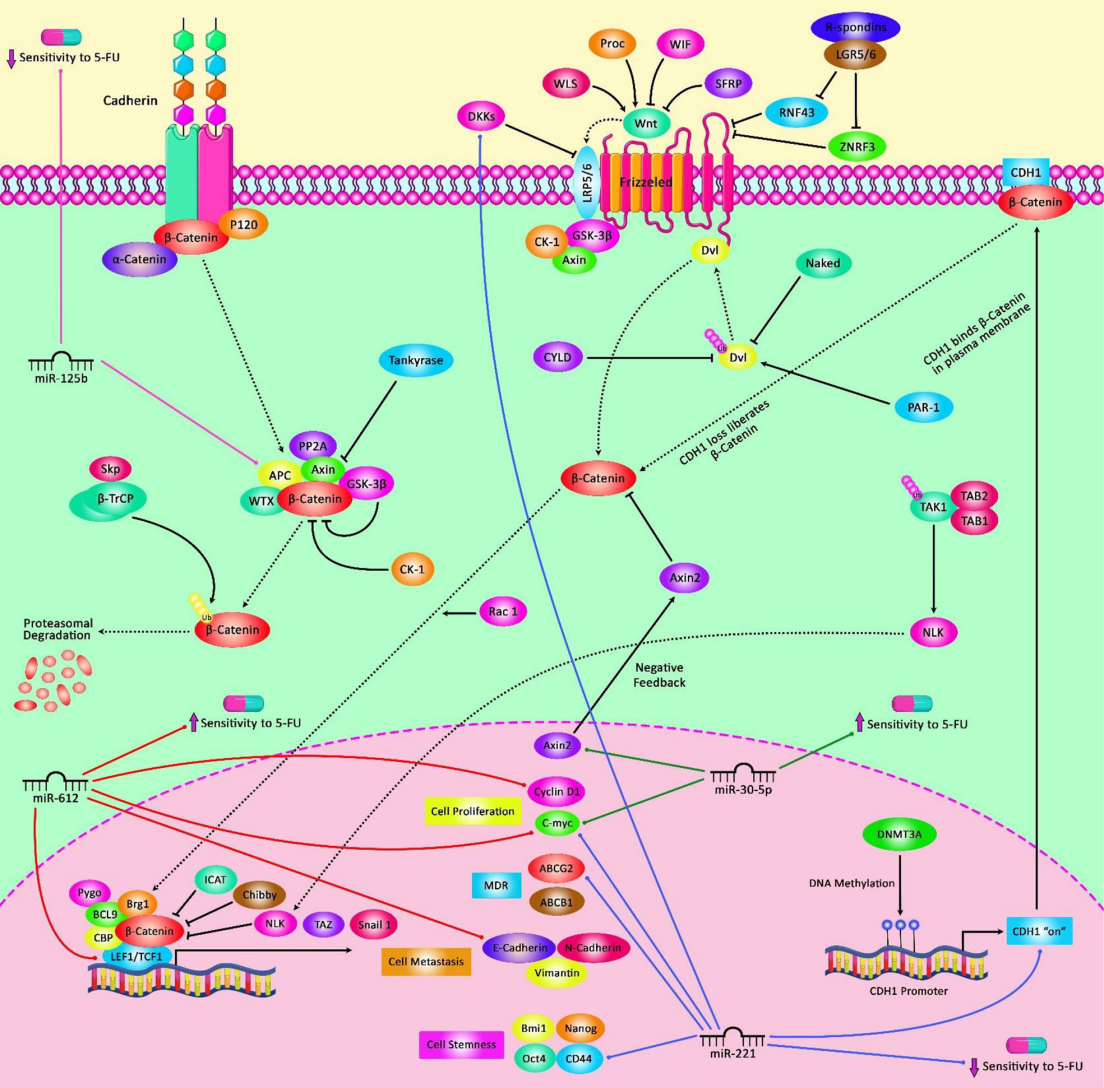
A schematic representation of the crosstalk between microRNAs and the Wnt/β-catenin pathway contributing in the modulation of 5-FU in the cancer cell. Mounting evidence has indicated that microRNAs dysregulation and the Wnt/β-catenin signaling pathway jointly drive the regulation of the sensitivity of tumor cells to 5-FU as a chemotherapeutic agent. As an illustration, miR-30-5p has been detected to function as a tumor suppressor via regulating the Wnt/β-catenin signaling cascade in colorectal cancer cells. miR-30-5p could downregulate the expression level of Wnt/β-catenin signaling target genes (MYC and Axin2) and the levels of β-catenin protein, thereby promoting the sensitivity of these target cells to 5-FU agent (48). Besides, miR-125b is a critical downstream mediator of the CXCL12/CXCR4 axis which could activate the Wnt/β-catenin signaling via targeting the APC gene and could play an effective role in enhancing invasion and 5-FU resistance by elevating autophagy in colorectal cancer cells (101).

In gastric cancer, miR-31 enhances sensitivity to 5-FU, decreases migration and invasion capacity, and surges the fraction of cells in G1/pre-G1 phase. These effects are possibly mediated through down-regulation of E2F6 and SMUG1 genes (97). On the other hand, miR-147 is an oncogenic miRNA in gastric cancer cells whose silencing has reduced cell proliferation and improved sensitivity of these cells 5-FU via modulating apoptotic pathway. Mechanistically, miR-147 down-regulates the expression of PTEN in gastric cancer cells and consequently modulates PI3K/AKT signaling pathway (98). Besides, the expression of miR-149 has been shown to be elevated in 5-FU-resistant gastric cancer cells compared with parental cells. miR-149 also enhances 5-FU resistance through reduction of TREM2 levels and regulation of β-catenin *in vivo* (99). Instead, the expression of miR-195 has been lower in 5-FU-resistant gastric cancer cells compared with the parental cells. Transfection of resistant cells with miR-195 has led to inhibition of HMGA1 expression and improvement of response to 5-FU (100). **Figure 1** demonstrates the role of several miRNAs in regulating the sensitivity of cancer cells to 5-FU via modulating the Wnt-β-catenin pathway which is a highly conserved cascade and is activated in the development of various human cancers like colorectal cancer.

**Table 3** provides a summary of experiments that reported the impact of miRNAs in the response of gastric cancer to 5-FU.

**Table 3:** Impact of miRNAs in the response of gastric cancer to 5-FU (ANT, adjacent normal tissue).

microRNA	Animal/Human	Assessed Cell line	Targets/Regulators	Function	Ref
miR-31	–	AGS, 293T, MKN-45	SMUG1, E2F6	MiR-31 could enhance sensitivity to 5-FU and decrease migration and cell invasion.	(97)
miR-147	mouse/human; 43 pairs of GC and ANTs	GES-1, AGS, SGC-7901, MKN-45, BGC-823, MGC-803	PTEN, PI3K/Akt	MiR-147 by targeting PTEN could enhance proliferation and trigger resistance to 5-FU.	(98)
miR-149	mouse/human: 20 pairs of GC and ANTs	AGS/5-FU, AGS	TREM2, β-catenin	MiR-149 by targeting TREM2 could contribute to resistance of 5-FU in GC cells via β-catenin signaling pathway.	(99)
miR-195	–	SGC-7901, AGS, SGC-7901/5-FU, AGS/5-FU	HMGA1	MiR-195 by targeting HMGA1 could enhance 5-FU sensitivity in GC cells.	(100)
miR-195-5p	12 gastric adenocarcinoma tissues	MKN28, MKN74	ZNF139	MiR-195-5p by targeting ZNF139 could reverse the multi−drug resistance of GC cells.	(102)
miR-197	–	SGC−7901, SGC7901/5−FU	MAPK1	MiR-197 by targeting MAPK1 could enhance sensitivity to 5-FU in CRC cells.	(103)
miR-204	mouse/human; 30 pairs of GC and ANTs	GES-1, AGS, SGC-7901, MKN-45, MGC-803, BGC-823	TGFBR2, EMT	MiR-204 by targeting TGFBR2 could inhibit proliferation, migration, and invasion in GC cells through EMT regulation.	(104)
miR-567	mouse/human; paired CRC and ANTs	GES-1, MKN45, BGC823, AGS, MGC803, BGC803, MKN28	PIK3AP1, PI3K/Akt	MiR-567 by targeting PIK3AP1 could inhibit tumor growth and reverse chemoresistance in GC cells via the PI3K/Akt signaling pathway.	(105)
miR-623	31 pairs of GC and ANTs	MKN-45, SGC-7901, BGC-823, MGC-803, GES-1	CCND1	MiR-623 by targeting CCND1 inhibits proliferation and enhances chemosensitivity to 5-FU.	(106)
miR-625	–	SGC7901, SGC7901/VCR, SGC7901/ADR	ALDH1A1	MiR-625 by directly targeting ALDH1A1 could reverse multidrug resistance and induce apoptosis in GC cells.	(107)
miR-1229-3p	mouse/human: 60 plasma samples of GC patients	HGC27, GFP-MKN45	SLC22A7, TS, DPD	MiR-1229-3p overexpression could induce chemoresistance of 5-FU and proliferation in GC cells.	(108)

A comprehensive study in esophageal cancer has frequent down-regulation of miR-29c in tumors and sera of these patients. Functionally, miR-29c has been shown to reverse 5-FU resistance in vitro and in vivo through direct interaction with the 3'UTR of FBXO31, resulting in suppression of FBXO31 and activation of p38 MAPK. Systemic administration of miR-29c has significantly enhanced response to 5-FU in xenograft models of esophageal cancer (109). In cervical cancer, miR-138/-135 can target FAK, enhance 5-FU sensitivity, and inhibit invasion and tumor growth (110). In pancreatic cancer cells, miR-221-3p, miR-486-5p, miR-21, and miR-320a influence response to 5-FU through targeting RB1, PTEN, and PDCD4 (111–114). Finally, in chronic myeloid leukemia (CML), miR-378 suppresses FUS1 expression, promotes cell proliferation, inhibits apoptosis, and confers resistance to 5-FU (115). **Table 4** provides an overview of researches that studied the role of miRNAs in the modulation of response to 5-FU in other types of cancer.

**Table 4:** Impact of miRNAs in the modulation of response to 5-FU in other types of cancer (ANT, adjacent normal tissue).

Cancer type	microRNA	Animal/Human	Assessed Cell line	Targets/Regulators	Function	Ref
Esophageal squamous cell carcinoma (ESCC)	miR-29c	mouse/human; multiple cohort studies including paired ESCC and ANTs and serum samples/ TCGA dataset	KYSE150FR, KYSE410FR, KYSE150, KYSE410	FBXO31, p38	MiR-29c by targeting FBXO3 could reverse chemoresistance to 5-FU in ESCC cells.	(109)
ESCC	miR-145	25 pairs of ESCC and ANTs	HEEC, TE-8, KYSE150, TE-1	REV3L	MiR-145 by targeting REV3L enhances 5-FU induced cell viability inhibition and cell apoptosis in ESCC cells.	(116)
ESCC	miR-338-5p	mouse/human; 72 pairs of ESCC and ANTs	KYSE410, KYSE150, KYSE270, T.Tn, 293T, KYSE410FR, KYSE150FR	Id-1	MiR-338-5p by targeting Id-1 could inhibit migration and invasion and reverse chemoresistance in ESCC cells.	(117)
Cervical Cancer	miR-138/-135	Mouse	HeLa	FAK	MiR-138/-135 by targeting FAK could increase chemosensitivity, inhibit invasion, and tumor growth.	(110)
Renal Cell Cancer (Rcc)	miR-381	–	786-O, HK-2	WEE1, Cdc2	MiR-381 by targeting WEE1 could trigger Cdc2 activation, mitotic catastrophe, and cell apoptosis and also enhance chemosensitivity in RCC cells.	(118)
Melanoma	miR-204-5p	mouse/human; 30 melanoma tissues and 30 benign nevi	A375, WM35, SK-MEL-5, SK-MEL-2, HEMa-LP	MMP9, Bcl-2	MiR-204-5p by targeting MMP9 and Bcl-2 could inhibit melanoma growth and resistance to 5-FU.	(119)
Gallbladder Carcinoma	miR-335	60 pairs of gallbladder carcinoma and ANTs	GBC-SD, SGC-996	MEF2D	MiR-335 by targeting MEF2D could inhibit cell growth and sensitize gallbladder carcinoma cells to 5-FU.	(120)
Cervical Cancer	miR-433	–	HeLa	TYMS	MiR-433 by negatively regulating TYMS could increase sensitivity for 5-FU and inhibit proliferation in HeLa cells.	(121)
Pancreatic Cancer (PaC)	miR-221-3p	–	PANC-1, PATU8988, 293TN, PATU8988/5-FU	RB1, EMT	MiR-221-3p by targeting RB1 could increase proliferation, migration, and invasion and also confer resistance for 5-FU in pancreatic cancer cells via the EMT signaling pathway.	(111)
PaC	miR-486-5p	mouse	PANC-1, MiaPaCa-2	PTEN, ERK, Akt	MiR-486-5p silencing could enhance cytotoxic effect of 5-FU.	(112)
PaC	miR-21	–	PATU8988, PANC-1, 293TN, PATU8988/5-FU	PTEN, PDCD4	MiR-21 by targeting PTEN and PDCD4 could increase resistance to 5-FU in pancreatic cancer cells.	(113)
PaC	miR-320a	–	PATU8988, PANC-1, 293TN, PATU8988/5-FU	PDCD4, EMT	MiR-320a by targeting PDCD4 could promote 5-FU resistance in human pancreatic cancer cells via EMT regulation.	(114)
CML	miR-378	59 bone marrow samples of CML and healthy controls	K562	FUS1, Nanog, OCT4, c-Myc	MiR-378 by repressing FUS1 could promote cell proliferation, inhibit apoptosis, and establish drug resistance to 5-FU in CML cells.	(115)

The expression pattern of several miRNAs that influence response to 5-FU is associated with the survival of patients with malignancies. Among oncogenic miRNAs, over-expression of miR-1229-3p in gastric cancer patients has been associated with shorter overall and relapse-free survival rates (108). On the other hand, down-regulation of several tumor-suppressive miRNAs such as miR-488, miR-145, and miR-199b has been associated with poor survival of cancer patients (43, 77, 92). The possible application of 5-FU-associated miRNAs as diagnostic markers has also been assessed showing the diagnostic power values of 0.807 and 0.77 for miR-1229-3p and miR-378 in gastric cancer and CML, respectively (108, 115). **Table 5** provides a summary of the importance of 5-FU-related miRNAs as diagnostic or prognostic markers in cancers.

**Table 5:** Diagnostic/prognostic roles of 5-FU-related miRNAs (OS, overall survival; RFS, relapse-free survival; DFS, disease-free survival).

Sample	Area Under Curve	Sensitivity	Specificity	Kaplan-Meier	Univariate/Multivariate Cox regression analysis	Ref
60 GC patients	0.807	73.7	80.5	High level of miR-1229-3p was associated with shorter OS and RFS rates.	A high level of miR-1229-3p was correlated with advanced TNM stages.	(108)
280 CRC patients	–	–	–	Low level of miR-488 was associated with shorter survival rate.	–	(77)
102 HCC patients	–	–	–	A low level of miR-145 was associated with a shorter survival rate.	A low level of miR-145 was correlated with lymph node metastasis and advanced TNM staging.	(92)
110 LARC patients	–	–	–	Low level of miR-199b was associated with shorter OS and RFS rates.	–	(43)
97 CRC patients	–	–	–	Low level of miR-552 was associated with shorter OS and DFS rates.	–	(81)
104 ESCC patients	–	–	–	Low level of miR-338-5p was associated with shorter survival rate.	–	(117)
TCGA dataset	–	–	–	Low level of miR-29c was associated with shorter OS rate.	–	(122)
59 CML patients (miR-378)	0.770	72.1	90.9	–	–	(115)
56 CRC patients	–	–	–	Low level of miR-329 was associated with shorter OS.	–	(72)
CRC patients from PROGgeneV2 database	–	–	–	Low level of miR-29c-3p was associated with shorter OS and MFS.	–	(47)
30 melanoma patients	–	–	–	Low level of miR-204-5p was associated with shorter survival.	–	(119)
152 CRC patients				A low level of miR-145 was associated with shorter survival.		(59)

## LncRNAs and Response to 5-FU

**Figure 2:**
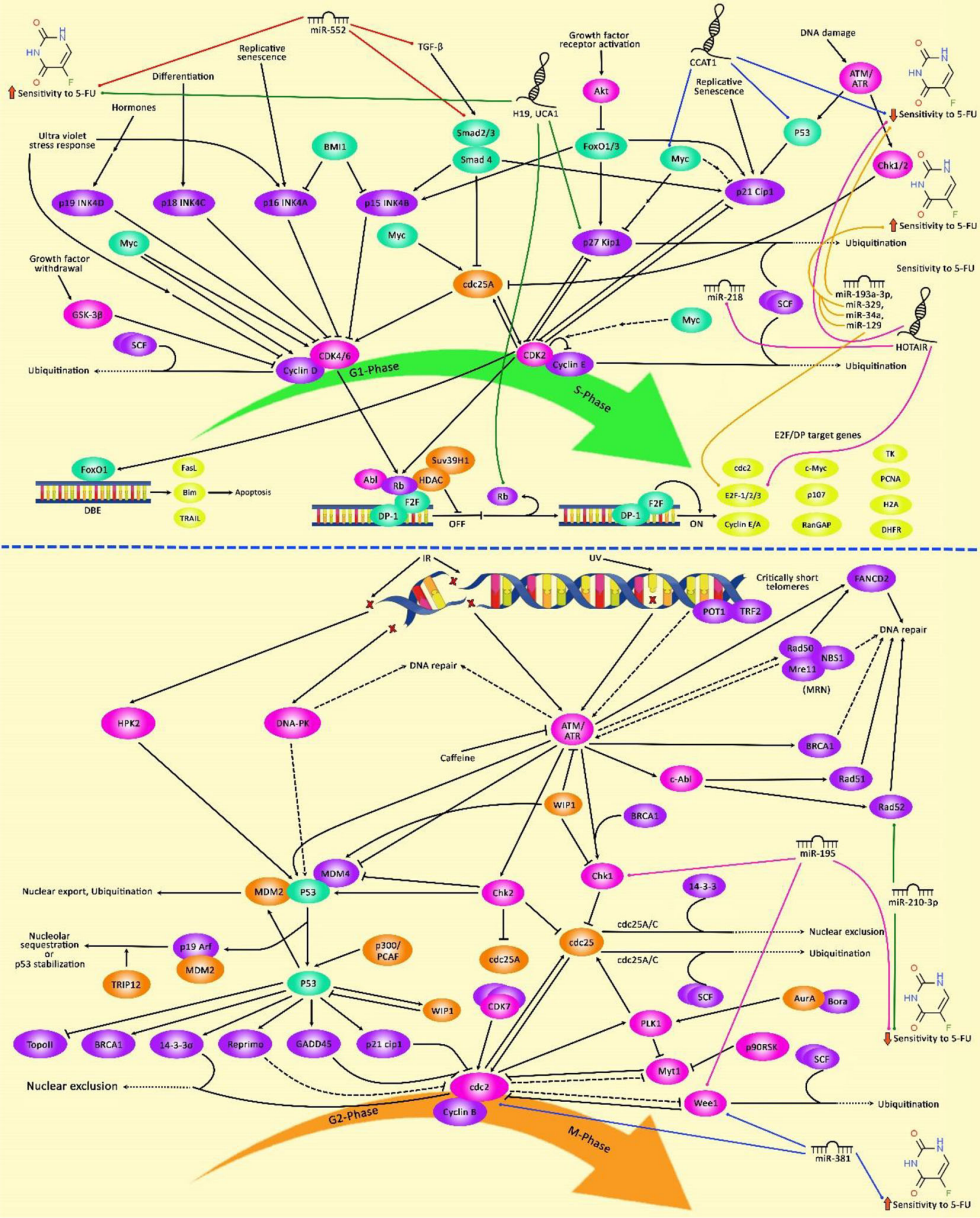
The schematic diagram of the effects of 5-FU on G1 and G2 phase cell cycle arrest in tumor cells through regulation by various non-coding RNAs. 5-fluorouracil has been highly applied for chemotherapy of gastrointestinal cancers and is known to affect the cell cycle and trigger apoptotic death of cancer cells. Non-coding RNAs have an important role in regulating cell cycle mechanisms via modulating the effects of 5-FU on the expression of G1/S and G2/M-related cell cycle regulators in tumor cells. LncRNA HOTAIR via downregulating the expression level of miR-218 and promoting the activation of NF-κB/TS signaling cascade could induce upregulation of the cell cycle transcription factor E2F-1, and thereby contributing to 5-FU Resistance and elevating enhanced colorectal cancer cell carcinogenesis (124). Besides, miR-381 via downregulation of the expression level of WEE1 and upregulation of the activity of Cdc2 results in alteration in cell cycle regulation which could potentiate the anti-tumor efficacies of 5-FU and abrogate G2/M cell cycle arrest in renal cancer cells (118). Additionally, miR-195 via directly targeting checkpoint kinase 1 (CHK1) and G2 checkpoint kinase WEE1 could desensitize colorectal cancer cells to 5-FU. This result demonstrates that miR-195 has a potential role in promoting the cell cycle via elevating G2/M transition after exposure to 5-FU (62).

Expression of HOTAIRM1 has been decreased in CRC tissues and cell lines, particularly in 5-FU resistant tissues and cells. In 5-FU resistant CRC cells, this lncRNA has been shown to suppress cell progression through acting as a competing endogenous RNA for miR-17-5p, thus enhancing the expression of BTG3 (123). HOTAIR is another lncRNA that induces resistance to 5-FU in CRC. This lncRNA down-regulates miR-218 level via an EZH2-related mechanism. HOTAIR silencing has suppressed cell viability and arrested cells in the G1-phase through enhancing miR-218 expression. VOPP1 is a functional target of this miRNA. Moreover, NF-κB signaling has been shown to be inhibited by HOTAIR via repression of miR-218 expression. Inactivation of NF-κB signaling by HOTAIR silencing has also partly reversed 5-FU resistance. Another route of participation of HOTAIR in resistance to 5-FU is the enhancement of TS expression (124). A high throughput assessment of transcriptome in 5-FU-resistant CRC cells and parental cells has revealed differential expression of more than 2,000 lncRNAs which have been enriched in Jak-STAT, PI3K-Akt, and NF-κB signaling pathways, emphasizing the role of these pathways in conferring resistance to 5-FU (125). **Table 6** summarizes the role of lncRNAs in the modulation of response to 5-FU in CRC. **Figure 2** illustrates that 5-FU-induced changes in cell cycle regulation of several cancer cells might be associated with an alteration of G1 and G2 target genes expression through the modulation by various non-coding RNAs.

**Table 6:** Role of lncRNAs in the modulation of response to 5-FU in colorectal cancer (ANT, adjacent normal tissue).

lncRNA	Human/Animal	Assessed Cell line	Targets/Regulators	Function	Ref
HOTAIRM1	athymic mice/human: 56 pairs of CRC and ANTs	HCT116, SW480, NCM460, HCT116/5-FU, SW480/5-FU	miR-17-5p, MRP1, MDR1, BTG3, E-cadherin, N-cadherin	HOTAIRM1 via sponging endogenous miR-17-5p/BTG3 axis could suppress cell progression in 5-FU resistant CRC cells.	(123)
HOTAIR	48 pairs of CRC and ANTs	HT29, SW480, FHC, HT29/5-FU, HCT116, SW620, SW1116, lovo, RKO, colon205	miR-218, VOPP1, TS, AKT, ERK, E2F-1, NF-kB	HOTAIR via suppressing miR-218 and activating NF-kB/TS signaling could contribute to 5-FU resistance.	(124)
uc010vzg.1, ENST00000468960	Microarray	HCT116, HCT116/5-FU	JAK/STAT, PI3K/AKT, NF-kB	Any change in lncRNA expression could be involved in 5-FU-based CRR in CRC cells.	(125)
PCAT6	73 pairs of CRC and ANTs	HCT116, HT‐29, SW620, SW480, DLD‐1, RKO, LoVo, 293T, CCD‐112CoN	miR‐204, HMGA2, PI3K/AKT	Overexpression of PCAT6 by inhibiting miR‐204 thereby promoting HMGA2/PI3K axis could enhance the chemoresistance of CRC cells to 5‐FU.	(126)
NEAT1	55 pairs of CRC and ANTs	FHC, HT29, HCT8, HCT116, SW480, SW620	miR-34a, Caspase-3, LC3 II/I, ULK1, Beclin-1, ATG9A, ATG4B, HMGB1	NEAT1 silencing could attenuate autophagy to elevate 5-FU sensitivity in CRC.	(127)
NEAT1	male BALB/c-nude mice/human; 30 pairs of CRC and ANTs	SW480, HCT116, NCM460	miR-150-5p, CPSF4, P-gp, GST-π	NEAT1 via the miR-150-5p/CPSF4 axis could regulate 5-Fu sensitivity in CRC.	(128)
ENST00000547547	–	HCT116, LoVo, LoVo/5-FU, HCT116/5-FU	miR-31, Bax, Bcl-2	ENST00000547547 via competitive binding to miR-31 could reduce the 5-FU resistance of CRC cells.	(129)
UCA1	119 pairs of CRC and ANTs	293T, HCT8, HCT116, HT29, LoVo, SW480,	miR-204-5p, CREB1, Bcl-2, RAB22A	UCA1 by inhibiting miR-204-5p could increase cell proliferation and 5-FU resistance in CRC.	(130)
XIST	268 pairs of CRC and ANTs	HT29, HCT116, FHC, HT29/5-FU, HCT116/5-FU	TS	XIST via promoting thymidylate synthase expression could inhibit 5-FU-induced CRC cell cytotoxicity.	(131)
TUG1	124 pairs of CRC and ANTs	HCT8Fu, HCT8, HCT116, SW1116	miR-197-3p, TYMS	TUG1 by acting as a ceRNA of miR-197-3p could mediate 5-FU resistance in CRC.	(132)
HAND2-AS1	nude mice/human; 27 pairs of CRC and ANTs	NCM460, HCT116, SW480, HCT116/5-FU, SW480/5-FU	miR-20a, PDCD4, Bax, Bcl-2, MMP2, MMP9	HAND2-AS1 by modulating miR-20a/PDCD4 axis could inhibit 5-FU resistance in CRC.	(133)
LINC00152	nude BALB/c mice/human; 108 pairs of CRC and ANTs	HCT8, HT29, LoVo, HCT116, SW480, SW620, 293T	miR-139-5p, NOTCH1	LINC00152 by inhibiting miR-139-5p could promote cell proliferation and confer 5-FU resistance in CRC.	(134)
H19	110 pairs of CRC and ANTs	HCT8, HCT8Fu, SW1116, 293T, HCT116, Lovo, HT29, SW480, SW620, CCD-18Co	Caspase-3, PARP, p62, LC3I/II, SIRT1	H19 by promoting SIRT1-mediated autophagy could confer 5-FU resistance in CRC.	(135)
CCAT1	BALB/c mice/human; 67 pairs of CC and ANTs	HCT 116, SW1417, HT-29, KM12, NCM460	γ-H2AX, p53, c-Myc	Downregulation of CCAT1 could enhance 5-FU sensitivity in CC cells.	(136)
H19, UCA1	–	HCT116, DLD1, SW480, HCT116/5-FU, DLD-1/5-FU, SW480/5-FU, HCT116/p, DLD-1/p, SW480/p	Rb, p27kip1	Overexpression of UCA1 and H19 could be involved in the impaired cell cycle in cells susceptible to 5-FU.	(137)

In addition to CRC, lncRNAs have fundamental roles in conferring resistance to 5-FU in other types of cancer, particularly gastric cancer and HCC. For instance, in gastric cancer cells, SNHG20 has been shown to mediate resistance to 5-FU via modulating the expression of miR-140-5p and subsequent up-regulation of its target gene NDRG3 (138). The role of PVT1 in the progression of this kind of cancer and induction of chemoresistance is mediated via up-regulation of the antiapoptotic gene Bcl2 (139). KRAL is a down-regulated lncRNA in HepG2/5-FU and SMMC-7721/5-FU cells compared with the corresponding parental cells. Up-regulation of KRAL has enhanced Keap1 expression. The resistance of these cells to 5-FU could be reversed through the inactivation of the Nrf2-dependent antioxidant pathway. KRAL serves as a sponge for miR-141 and subsequently restores Keap1 expression (140). NR2F1‐AS1 is involved in the modulation of response to 5-FU in breast cancer cells. This lncRNA increases IGF‐1 levels *via* sponging miRNA‐338‐3p and then activates IGF‐1R and ERK pathways (141). TMPO-AS1 is an over-expressed lncRNA in ovarian cancer tissues and SKOV3 cells. This lncRNA regulates TMEFF2 via sponging miR-200c. Moreover, it activates the PI3K/Akt signaling pathway. TMPO-AS1 silencing has suppressed epithelial–mesenchymal transition (EMT), invasiveness, migration and 5-FU resistance in ovarian cancer cells (142). **Table 7** summarizes the role of lnRNAs in modulation of response to 5-FU in other cancers.

**Table 7:** Role of lncRNAs in the modulation of response to 5-FU in other cancers (ANT, adjacent normal tissue).

Cancer type	lncRNA	Human/Animal	Assessed Cell line	Targets/Regulators	Function	Ref
Gastric Cancer (GC)	SNHG20	GC tissues (n = 408), normal stomach tissue (n = 211)	BGC−823, AGS	miR−140−5p, NDRG3	SNHG20 via miR−140−5p/NDRG3 axis could contribute to 5−FU resistance in GC.	(138)
GC	PVT1	Nod/SCID mice/human; normal (n = 35), GC (n = 229)	SGC-7901,	Bax, Bcl-2, Caspase-3	PVT1 via increasing Bcl-2 could mediate 5-FU resistance in GC.	(139)
GC	HOTAIR	168 pairs of GC and ANTs	–	–	HOTAIR could be considered as a biomarker for patients with advanced GC.	(143)
Hepatocellular Carcinoma (HCC)	KRAL	30 pairs of HCC and ANTs	HepG2, HepG2/5-FU, SMMC-7721, SMMC-7721/5-FU	Keap1, miR-141	KRAL by acting as a ceRNA against miR-141 could reverse 5-FU resistance in HCC cells.	(140)
Breast Cancer (BC)	SNORD3A	female BALB/c athymic nude mice/human; 26 pairs of BC and ANTs	MCF10A, MCF-7, MDA-MB-231, T47D, SKBR3, ZR7530, BT549, HCC1937, BT474, 293T	GFP, UMPS, Meis1	SNORD3A by sponging miR-185-5p to enhance UMPS could sensitize BC cells to 5-FU.	(144)
Esophageal cancer (EC)	LINC00261	BALB/c nude mice/human; EC (n = 162), normal tissue (n = 11)	Het-1A, KYSE150, Eca109, TE-1, TE-5, TE-1/5-FU	DPYD	LINC00261 by mediating methylation-dependent repression of DPYD could induce chemosensitization to 5-FU in EC.	(145)
EC	LINC01270	male nude mice, 42 pairs of EC and ANTs	TE-13/5-FU, Eca-109, KYSE450, TE-13, EC109, TE-11	GSTP1, DNMT3B, MMP2	Silencing of LINC01270 by mediating GSTP methylation could enhance chemosensitivity to 5-FU and inhibit EC progression.	(141)
EC	HOTAIR	nude mice/human, 70 pairs of EC and ANTs	KYSE150, EC109, TE-1, HEEC, TE-1/5-FU	MTHFR	HOTAIR by attenuating the promoter hypermethylation of the MTHFR could sensitize EC cells to 5-FU.	(146)
ESCC	LINC01419	nude mice/human; 38 pairs of ESCC and ANTs, GSE21362 database	Het-1a, KYSE70, KYSE450, EC109, EC9706	GSTP1	Overexpression of LINC01419 via promoting GSTP1 methylation could diminish the sensitivity of ESCC cells to 5-FU.	(147)
Ovarian cancer	TMPO-AS1	BALB/C nude mice/human; GEO database	HOSEpiC, SKOV3, SKOV3/5-FU	miR-200c, TMEFF2, PI3K/AKT	Knockdown of TMPO-AS1 via the miR-200c/TMEFF2 axis and disrupting the PI3K/Akt signaling could inhibit the invasion, metastasis, and drug resistance of OC cells.	(142)
Pancreatic ductal adenocarcinoma (PDAC)	DGCR5	30 pairs of PDAC and ANTs	HPDE6, PANC-1, SW1990,BxPC-3, HPAC, MIAPaCa-2, HPDE6/5-FU, PANC-1/5-FU	E-cadherin, Twist, Vimentin, miR-320a,	Overexpression of DGCR5 via targeting miR-320a/PDCD4 axis could promote 5-FU resistances of PDAC cells.	(148)

**Figure 3:**
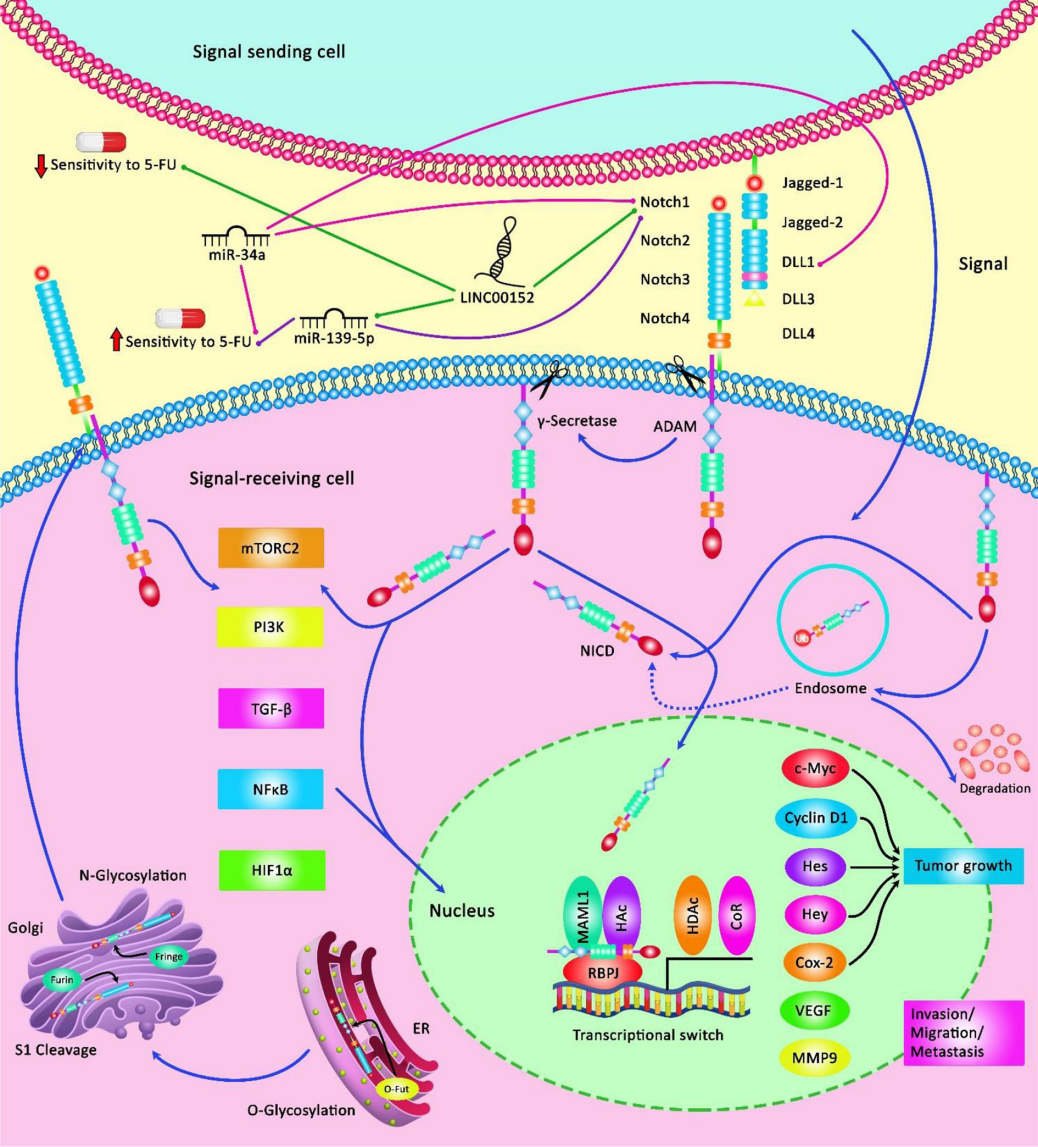
A schematic illustration of the Notch signaling pathway involved in the regulation of response of cancer cells to 5-FU via various non-coding RNAs. Notch signaling cascade is involved in the various processes of normal morphogenesis, such as cell growth, apoptosis, as well as the acquisition of drug resistance. LINC00152 could elevate tumor cell migration and invasion, and confer 5-FU resistance in colorectal cancer via modulating the expression level of NOTCH1 through sponging miR-139-5p and downregulating its function from enhancing CRC development (134). Additionally, miR-34a acts as a tumor suppressor and could directly downregulate the expression level of DLL1 as a ligand of the Notch signaling cascade, and thereby could inhibit tumor growth under 5-FU treatment by promoting chemosensitivity to this agent (51).

In gastric cancer, Kaplan–Meier analysis has demonstrated that patients with over-expression of PVT1 do not benefit from 5-FU containing chemotherapeutic regimens. However, therapeutic regimens containing no 5-FU have been shown to increase the first progression survival and overall survival of this group of patients suggesting the role of PVT1 as a predictor of resistance to 5-FU treatment (139). Additional studies in CRC have shown the importance of expression levels of several lncRNAs namely HOTAIR, PCAT6, UCA1, XIST, TUG1, HAND2-AS1, LINC00152, and H19 in the determination of patients’ prognosis (**Table 8**). **Figure 3** depicts the regulation of the efficacy of 5-FU-based chemotherapy in cancer cells via multiple non-coding RNAs through the Notch signaling cascade.

**Table 8:** Prognostic roles of 5-FU-related lncRNAs (ANTm adjacent normal tissue; OS, overall survival; RFS, relapse-free survival).

Sample	Kaplan–Meier	Multivariate Cox regression analysis	Ref
48 pairs of CRC and ANTs	Higher expression of HOTAIR was related to lower OS and RFS rates.	Higher expression of HOTAIR was related to tumor size, distant metastasis, and tumor differentiation.	(124)
73 pairs of CRC and ANTs	Higher expression of PCAT6 was related to a lower OS rate.	Higher expression of HOTAIR was related to TNM stage, tumor differentiation, and lymph node metastasis.	(126)
119 pairs of CRC and ANTs	Higher expression of UCA1 was related to a lower OS rate.	Higher expression of UCA1 was related to tumor size and lymph node invasion.	(130)
268 pairs of CRC and ANTs	Higher expression of XIST was related to lower OS and RFS rates.	Higher expression of XIST was related to TNM stage and distant metastasis.	(131)
124 pairs of CRC and ANTs	Higher expression of TUG1 was related to lower RFS rate.	Higher expression of TUG1 was related to the depth of the tumor.	(132)
27 pairs of CRC and ANTs	Lower expression of HAND2-AS1 was related to lower OS rate.	–	(133)
108 pairs of CRC and ANTs	Higher expression of LINC00152 was related to lower OS and DFS rates.	Higher expression of LINC00152 was related to the tumor stage.	(134)
110 pairs of CRC and ANTs	Higher expression of H19 was related to lower RFS rate.	–	(135)
168 pairs of GC and ANTs	Higher expression of HOTAIR was related to lower OS rate.	Higher expression of HOTAIR was related to tumor size and TNM stage.	(143)

## Application of the CRISPR/Cas9 System With the Aim of Overcoming 5-FU Resistance in Human Cancer Cells

Accumulating evidence revealed that the CRISPR-Cas9 gene-editing tool can be considered as a potential approach in order to promote sensitivity to chemotherapeutic agents. Due to the reason that gene mutation plays a remarkable role in developing drug resistance in tumor cells, CRISPR-Cas9 can be employed as an effective gene manipulation system with regards to permanently removing genes and attenuating resistance to cancer chemotherapy (149–151). Furthermore, this applicable method can be applied effectively and has a great advantage compared to other gene-editing technologies such as siRNAs, ZFNs, and TALENs in manipulating target genes involved in the chemotherapy resistance (152–154). Clinical evidence demonstrated that cartilage oligomeric matrix protein (COMP) has a substantial part in tumorigenesis, proliferation, and invasion of colon cancer cells. Utilizing the CRISPR/Cas9 system, it has been possible to create COMP-knockout cells *via* lentiviral vectors which could, in turn, enhance the sensitivity of tumor cells to 5-FU and suppress PI3K/Akt/mTOR/p70S6K pathway (155). In addition, Lobo et al. figured out that employing the CRISPR/Cas9 gene-editing tool for manipulation of CD44 in addition to Phosphorodiamidate Morpholino oligomers (PMOs) can promote cisplatin and 5-FU sensitivity in gastric tumor cells (156). Furthermore, due to the association of GPRC5a with a worse prognosis, the CRISPR/Cas9 was employed to knock-out the expression level of this target gene to reduce proliferation and migration ability of tumor cells and inhibit resistance to oxaliplatin, 5-FU, and gemcitabine in pancreatic cancer cells (157). Moreover, current research indicated that upregulation of MUC5AC could widely affect colorectal cancer chemotherapy response via overexpression of β-catenin and its target genes CD44 and Lgr5 as well as suppression of p53 and its target gene p21, which is frequently associated with aggressiveness of colorectal cancer cells. RNA interference and CRISPR/Cas9 systems were utilized to knock-out the expression of MUC5AC in tumor cells thereby enhancing the sensitivity of cancer cells to 5-FU and oxaliplatin (158). With the emergence of the CRISPR-Cas9, experimentations in the field of drug resistance in various human cancers have been advanced greatly. A summary of clinical researches related to the knockout of various genes causing 5-FU resistance in several human cancer cells via the CRISPR/Cas9 gene-editing tool is demonstrated in **Table 9**. Although these studies have targeted mRNA coding genes, they show the feasibility of targeting certain transcripts and the significant effects of these methods in sensitization of neoplastic cells to 5-FU. Similar strategies targeting lncRNAs/miRNAs would have similar effects on cancer cells.

**Table 9:** Pre-clinical studies employing CRISPR/Cas9 to recognize the role of various genes in response to 5-FU treatment.

Cancer	Target	*In vitro*	Cell line	Animal	*In vivo*	CRISPR	Vector	Other gene-editing methods	Treatment	Signaling	Effect	Ref
Colorectal cancer (CRC)	MUC5AC	**+**	HCT-8, LS174T	5–6-week-old athymic nude mice	**+**	Knockout (targeting exon 2)	Lentiviral	siRNA	5-fluorouracil (5-FU), Oxaliplatin	CD44/β-catenin/p53/p21	Sensitized the cells	(158)
CRC	SNHG15	+	LoVo	6–7-week-old female BALB/c-Rag2/−IL2cc/immunodeficient mice	+	Knockout (deleting the region between exon 3 and 5)	plasmid	siRNA	5-FU	**–**	Sensitized the cells	(159)
CRC	CYSLTR1	+	HT-29, HT-29-R	**−**	**−**	Knockout	Plasmid	**–**	5-FU	LTD_4_/CysLT_1_R	Sensitized the cells	(160)
CRC	COMP	+	HEK 293T, LoVo, SW1116	4-week-old male BALB/c nude mice	+	Knockout (targeting exon 10)	Lentiviral	**–**	5-FU	PI3K/Akt/mTOR/p70S6K	Sensitized the cells	(155)
CRC	BAG3	+	HCT-116	**−**	**−**	Knockout	Lentiviral	**–**	5-FU	JAK/Stat, ERK/MAP, AMPK PTEN, PI3K/AKT	Sensitized the cells	(161)
CRC	LINC01021	+	HCT116	**−**	**−**	Deletion of promoter sequences (MER61C LTR element)	Plasmid	siRNA	5-FU, Doxorubicin	**–**	Sensitized the cells	(162)
CRC	FoxO3A	+	HCT116	**−**	**−**	Knockout (targeting exon 2)	Plasmid	siRNA	5-FU, Irinotecan, Cisplatin, Etoposide	MEK/ERK, AMPK	Sensitized the cells	(163)
Gastric cancer (GC)	cd44v6	+	MKN45, GP202	**−**	**−**	Editing (targeting Exon-v6 Splice-Sites)	Plasmid	PMOs	5-FU, Cisplatin	**–**	Sensitized the cells	(164)
GC	GSDME	+	MKN-45, SGC-7901	**−**	**−**	Knockout	Plasmid	siRNA	5-FU	**–**	Sensitized the cells	(165)
Metaplasia	DDIT4	+	MGC-803	**−**	**−**	Knockout	Plasmid	**–**	5-FU	mTORC1	Sensitized the cells	(166)
Myeloid malignancies (MDS, AML)	ASXL1	+	U937	**−**	**−**	Frameshift mutation (targeting a specific site (nt1010-1031) of exon 8)	Plasmid	**–**	5-FU	**–**	Sensitized the cells	(167)
Nasopharyngeal carcinoma (NPC)	EBV DNA	+	C666-1, HEK293M81	**−**	**−**	Editing (targeting EBNA1, OriP, and W repeats)	Plasmid	**–**	5-FU, Cisplatin	**–**	Sensitized the cells	(168)
Oesophageal adenocarcinoma (OAC)	TP53	+	OE33, OE19, H1299, HEK293T, JH-EsoAd1, FLO-1, OACM5.1, Eso26, SKGT4, OACP4C, TE7, OANC1, NES	6-week-old female nude (Eso26 and OE19), NOD-SCID IL-2RγKO mice (FLO-1)	+	Knockout (targeting exon 5)	Lentiviral	siRNA	5-FU, Cisplatin, Epirubicin	P53	Sensitized the cells	(169)
Pancreatic cancer (PaCa)	GPRC5a	+	MIA PaCa-2, TB32047	**−**	**−**	Knockout	Plasmid	**–**	5-FU, Gemcitabine, Oxaliplatin	**–**	Sensitized the cells	(157)
–	uPAR	+	HCT8/T, KBV200	**−**	**−**	Knockout (targeting exon 2)	Lentiviral	**–**	5-FU, Cisplatin, Docetaxel, Doxorubicin	**–**	Sensitized the cells	(170)

## Effect of Histone Deacetylase Inhibitors in Combination With5-FU on Promoting the Chemotherapeutic Efficacy in Multiple Human Cancers

Accumulating evidence has demonstrated that tumorigenesis not only occurs by a genetic mutation, but it also could be triggered via epigenetic alteration processes. Modification of histones by acetylation has an important part in epigenetic modulation of gene expression which is regulated by both histone acetyltransferases (HAT) and histone deacetylases (HDAC) (171). Since dysregulation of histone acetylation is a hallmark of cancer in some cases, thereby employing HDAC inhibitors could shed novel insights into regulatory mechanisms of transcription as well as inducing differentiation and apoptosis. HDAC inhibitors are potential anticancer drugs because of their ability to induce tumor cell differentiation, cell cycle arrest, and cell death, attenuate angiogenesis, reverse transformed cell morphology, and regulate immune response (172). Importantly, the combination of HDAC with 5-FU can elevate the efficacy of this agent in tumor cells. The HDAC inhibitor SAHA has a key role in promoting sensitivity to 5-FU and irinotecan via triggering proapoptotic signals in hepatocellular carcinoma cells through overcoming MDR-mediated drug efflux, suppressing SN-38 glucuronidation and synchronization of the cell cycle by upregulation of CDK-inhibitor p21cip1/waf1 (173). Additionally, Minegaki et al. have demonstrated that co-administration of 5−FU with VPA or SAHA as an inhibitor of histone deacetylases could downregulate the expression level of TS in 5−FU−resistant MDA−MB−468 breast cancer cells, and thereby promoting the sensitivity of both 5-FU-sensitive and −resistant breast cancer cells to 5-FU chemotherapy (174). Moreover, another research has illustrated that the combination of HDAC inhibitor depsipeptide and 5-FU could significantly enhance the sensitivity of colon cancer cells to chemotherapy. This sensitization of tumor cells could occur via triggering cell cycle arrest caused by overexpression of p21 (CDKN1A), modulating apoptosis represented by caspase-3/7 activation as well as regulating the expression level of MHC class II (175). In addition, Hamam et al. have detected that the effect of 5−FU against colon tumor cells could be promoted remarkably by the combination treatment with CUDC−907, a dual HDAC, and PI3K inhibitor. This could in turn lead to upregulation of apoptotic processes and necrosis, as well as enhancing polyploidy (176). Besides, Tan et al. have shown that the HDAC6 selective inhibitor ACY1215 could play an effective role in inhibiting colon cancer cell growth, migration, invasion, and triggering apoptosis in colon cancer cells, and thereby elevating the efficacy of 5-FU (177). On the other hand, another study showed that upregulation of HDAC4 could modulate TGFβ signaling cascade via reducing the expression levels of SMAD4, SMAD6, BMP6, iID1, TGFβ2, and TGFβ3 in breast cancer cells. HDAC4 could also regulate the expression of SMAD4 via histone h3 deacetylation which could in turn augment MDA-MB-231 cell resistance to 5-FU (178). The effects of epigenetic regulators on the expression of lncRNAs/miRNAs which are linked with cellular response to 5-FU have been less studied.

## The Role of Autophagy in 5−FU Treatment in Multiple Human Cancers

Multidrug resistance (MDR) could occur mostly after long−term chemotherapy, leading to tumor recurrence. Autophagy, a self−degradative mechanism, generally occurs during the process of resistance to chemotherapy. Autophagy can enhance the MDR and protection of tumor cells from these drugs. Autophagy induced by anticancer agents could also trigger upregulation of apoptotic signaling pathways in MDR cells, simplifying MDR reversal (179–181). Accumulating evidence illustrated that suppression of autophagy by either pharmacological procedures or through regulatory gene silencing enhances 5−FU−induced tumor cell death. Furthermore, autophagy could have a pro−death role which may modulate cell death in various tumor cells to trigger apoptosis pathways. Therefore, autophagy could be a target to improve the sensitivity of multiple cancer cells to 5−FU (20). Zhang et al. have illustrated that a combination of 5-FU and β-Elemene could play an effective role in promoting the sensitivity of p53-deficient colorectal cancer cells to 5-FU via modulation pro-death autophagy by promoting the formation of autophagosome (182). Furthermore, another research has demonstrated that psilostachyin-A can attenuate 5-FU resistance in liver carcinoma via triggering autophagy in these cells. Psilostachyin-A could cause the enhancement of the autophagosomes via upregulating the expression levels of LC3B-II and Beclin-1 in the HepG2 cells. This could also induce G2/M arrest of the tumor cells through declining of cyclin B1 and CDK1 expression as well as suppressing the MAPK/ERK signaling cascade, and thereby inhibiting proliferation and invasion of the HepG2 cells to the large extent (183). Besides, Zhang et al. have detected that whilst autophagy could be activated by treatment of human colon carcinoma cells with 5-FU, treatment of these cells with curcumin followed by the 5-FU treatment could considerably attenuate autophagy activation and promote the cytotoxicity of this chemotherapeutic drug. This could occur via the alteration in LC3II/LC3I, beclin-1, and p62 expression levels in cancer cells which could, in turn, contribute to the downregulation of Akt/mTOR as well as AMPK/ULK1 signaling cascades in HCT116 cells (184). Furthermore, Yu et al. have represented that miR-125b could enhance 5-FU resistance in colorectal cancer cells via promoting cell autophagy. miR-125b remarkably upregulates the expression levels of beclin-1 and cleaved LC3B-II which could, in turn, play an important role in triggering autophagy and reducing the sensitivity of cancer cells to 5-FU. miR-125b was increased by the activation of the CXCL12/CXCR4 axis, and thereby miR-125b could significantly augment EMT. Inhibition of this miRNA may be a suitable approach to attenuate the development of chemoresistance in tumor cells which could play a critical role in the regulation of autophagy (101). Similarly, several miRNAs/lncRNAs that affect response to 5-FU modulate autophagy in the neoplastic cells.

## Discussion

A vast body of literature has revealed the impact of non-coding RNAs in the determination of the response of cancer cells to 5-FU. CRC and HCC are the most assessed cancer types in this regard possibly because of the vast application of this chemotherapeutic agent in these types of cancer. Yet, the influence of non-coding RNAs in the modulation of response to 5-FU has been mostly assessed *in vitro* needing confirmation in animal models and human subjects. Autophagy has been identified as the main route of the function of non-coding RNAs in the determination of the response of cancer cells to 5-FU. Moreover, the influence of non-coding RNAs on apoptotic-related pathways also affects the response of cancer cells to 5-FU. EMT is also influenced by these non-coding RNAs. The latter function of lncRNAs and miRNAs is consistent with the observed association between EMT and acquired resistance to 5-FU in cancer cells (185).

It is crucial to emphasize that the results of *in vitro* studies regarding the role of non-coding RNAs in the modulation of response to 5-FU should be verified in animal models as well as human subjects. Although the results of these three types of studies are mostly consistent, there are few examples of inconsistency. For instance, while miR-23a antisense has enhanced the activation of the caspases-3 and -7 and increased the 5-FU-associated apoptosis in CRC cells, this approach has not improved the anticancer impact of 5-FU in the xenograft model of CRC (42).

Exosome-mediated transfer of non-coding RNAs particularly miRNAs is implicated in conferring chemoresistance at a wide distance from the original cells. Moreover, these cell-free particles can modulate several cells in the tumor microenvironment in favor of tumor progression. On the other hand, it is possible to take advantage of exosomes as vehicles for the specific transfer of anti-cancer agents to cancer cells. A successful example of the latter function of exosomes has been provided by simultaneous delivery of 5-FU and miR-21 inhibitor oligonucleotide to Her2 expressing cancer cells via engineered exosomes (186).

LncRNAs mostly affect the response of cancer cells to 5-FU through the modulation of the expression of miRNAs. HOTAIRM1/miR-17-5p, HOTAIR/miR-218, PCAT6/miR‐204, NEAT1/miR-34a, NEAT1/miR-150-5p, ENST00000547547/miR-31, UCA1/miR-204-5p, TUG1/miR-197-3p, HAND2-AS1/miR-20a, KRAL/miR-141, SNHG20/miR-140-5p and LINC00152/miR-139-5p are examples of the roles of lncRNAs in sponging miRNAs in the context of 5-FU resistance. A number of lncRNAs such as XIST have been shown to directly influence the expression of 5-FU-related genes such as TS.

In spite of the comprehensive data about the effect of miRNAs and lncRNAs in the modulation of response of cancer cells to 5-FU, therapeutic efforts are scarce in this field. An important study in this field has shown the significant effect of systemic administration of miR-29c in the enhancement of response to 5-FU in the xenograft model of esophageal cancer (109). Conduction of similar studies using mimics or antamiRs for other miRNAs is a necessity for translation of the valuable basic science in this filed into clinical use.

Finally, the expression signature of miRNAs and lncRNAs which confer resistance to 5-FU has been associated with the survival of patients with different types of cancer. This observed association is not necessarily related to the role of these transcripts in chemoresistance particularly in cancer patients who have not been treated with this agent. Instead, it might merely reflect the oncogenic or tumor-suppressive effects of these transcripts.

### Perspectives and Future Directions

Generally, this review provides convincing evidence for the role of miRNAs and lncRNAs as biomarkers of response to 5-FU treatment in a variety of solid tumors, especially in colorectal cancer cells. Patients with gastrointestinal cancer become resistant to 5-FU because of the aberrant expression of certain genes. This event is a prevalent phenomenon in clinical practice. Modulation of expression levels of miRNAs or lncRNAs may be a suitable approach to sensitize tumor cells to 5-FU treatment via modulating multiple biological signaling pathways like Hippo/YAP, Wnt/β-catenin, Hedgehog, NF-kB, and Notch cascades. There is an increasing interest in targeting miRNAs as well as lncRNAs in various kinds of cancers that are treated by 5-FU. However, due to the wide range of miRNAs and lncRNAs regulating the response to 5-FU and their aberrant expression in multiple cancers, it is required to characterize the most clinically relevant non-coding RNAs in these malignancies. Therefore, researchers should systematically investigate the correlations between genes, pathways, and drug sensitivity to find direct causal effects. Besides, the research procedures recently utilized are mainly phenotype-based, like *in vitro* cell proliferation, migration and invasion, and *in vivo* mouse specimens. To find practical strategies, novel gene editing systems such as the CRISPR/Cas9 method should be applied to figure out the biological role of various target genes as well as non-coding RNAs in human cancers. Additionally, the enhancement of human primary cell models and patient-derived tumor xenograft (PDX) animal models may also play a key role in scrutinizing the role of non-coding RNAs and improving non-coding RNA-targeting techniques. We also suggest that non-invasive liquid biopsies such as circulating tumor DNA (ctDNA) and circulating tumor cells (CTCs) should be employed to identify factors that are explicitly accompanied with 5-FU sensitivity and/or adverse reactions (187, 188). These methods can help in the reduction of ineffective therapies and overdose as well as attenuating toxic side effects of 5-FU. Moreover, based on sufficient experimental data, we propose that the procedure of downregulating autophagy by either pharmacological methods or via silencing genes involved in the autophagy could also be considered as effective adjunctive therapy to improve the sensitivity of tumor cells to 5-FU. Besides, we propose that epigenetic processes such as modification of histones by acetylation can influence response to 5-FU. The obtained information from these studies will guide the advancement of precision medicine in the upcoming future.

## Author Contributions

MT and SG-F supervised the study, wrote the draft, and edited the submission. HS, AA, FT, FF, and SJ performed the data collection and designed the tables and figures. All of the authors contributed equally and fully aware of submission. All authors contributed to the article and approved the submitted version.

## Conflict of Interest

The authors declare that the research was conducted in the absence of any commercial or financial relationships that could be construed as a potential conflict of interest.

## Abbreviations

5-FU, 5-fluorouracil; SCC, squamous cell carcinoma; ADC, adenocarcinoma; VEGF, vascular endothelial growth factor; EGFR. epidermal growth factor receptor; TS, thymidylate synthase; FdUMP, Fluorodeoxyuridine monophosphate; FdUTP, fluorodeoxyuridine triphosphate; FUTP, fluorouridine triphosphate; HMGB1, High Mobility Group Box 1; MTHFR, methylenetetrahydrofolate reductase; DPD, Dihydropyridine dehydrogenase; dTTP, deoxythymidine triphosphate; UTP, uridine triphosphate; TK, thymidine kinase; DPYD, dihydropyrimidine dehydrogenase; DHFU, 5,6-dihydro-5-fluorouracil; FBAL, α-fluoro-β-alanine; CYPs, Cytochromes P450; CDHP, 5-chloro-2, 4-dihydroxypyridine; DMEs, drug-metabolizing enzymes; AC, acid ceramidase; CRC, colorectal cancer; BTG1, B-cell translocation gene 1; HCC, Hepatocellular carcinoma; CML, chronic myeloid leukemia; ESCC, Esophageal squamous cell carcinoma; Rcc, Renal Cell Cancer; PaC, Pancreatic Cancer; CHK, 1checkpoint kinase 1; EMT, epithelial–mesenchymal transition; GC, Gastric Cancer; PDAC, Pancreatic ductal adenocarcinoma; COMP, cartilage oligomeric matrix protein; NPC, Nasopharyngeal carcinoma, OAC, Oesophageal adenocarcinoma; HAT, histone acetyltransferases; HDAC, histone deacetylases; CDKN1A, cell cycle arrest caused by overexpression of p21; MDR, Multidrug resistance; PDX, patient-derived tumor xenograft; ctDNA, circulating tumor DNA; CTCs, circulating tumor cells.

## References

[1] Miura K Kinouchi M Ishida K Fujibuchi W Naitoh T Ogawa H. 5-fu metabolism in cancer and orally-administrable 5-fu drugs. Cancers (Basel) (2010) 2(3):1717–30. 10.3390/cancers2031717 PMC3837334 24281184

[2] Vermorken JB Remenar E van Herpen C Gorlia T Mesia R Degardin M. Cisplatin, Fluorouracil, and Docetaxel in Unresectable Head and Neck Cancer. New Engl J Med (2007) 357(17):1695–704. 10.1056/NEJMoa071028 17960012

[3] Argilés G Tabernero J Labianca R Hochhauser D Salazar R Iveson T. Localised colon cancer: ESMO Clinical Practice Guidelines for diagnosis, treatment and follow-up. Ann Oncol (2020) 31(10):1291–305. 10.1016/j.annonc.2020.06.022 32702383

[4] Chen Y Ye J Zhu Z Zhao W Zhou J Wu C. Comparing Paclitaxel Plus Fluorouracil Versus Cisplatin Plus Fluorouracil in Chemoradiotherapy for Locally Advanced Esophageal Squamous Cell Cancer: A Randomized, Multicenter, Phase III Clinical Trial. J Clin Oncol (2019) 37(20):1695–703. 10.1200/JCO.18.02122 PMC6638596 30920880

[5] Sakai M Sohda M Saito H Kuriyama K Yoshida T Kumakura Y. Docetaxel, cisplatin, and 5-fluorouracil combination chemoradiotherapy for patients with cervical esophageal cancer: a single-center retrospective study. Cancer Chemother Pharmacol (2019) 83(6):1121–6. 10.1007/s00280-019-03835-0 30972455

[6] Heidelberger C Chaudhuri NK Danneberg P Mooren D Griesbach L Duschinsky R. Fluorinated pyrimidines, a new class of tumour-inhibitory compounds. Nature (1957) 179(4561):663–6. 10.1038/179663a0 13418758

[7] Hurwitz H Fehrenbacher L Novotny W Cartwright T Hainsworth J Heim W. Bevacizumab plus irinotecan, fluorouracil, and leucovorin for metastatic colorectal cancer. N Engl J Med (2004) 350(23):2335–42. 10.1056/NEJMoa032691 15175435

[8] Van Cutsem E Joulain F Hoff PM Mitchell E Ruff P Lakomý R. Aflibercept Plus FOLFIRI vs. Placebo Plus FOLFIRI in Second-Line Metastatic Colorectal Cancer: a Post Hoc Analysis of Survival from the Phase III VELOUR Study Subsequent to Exclusion of Patients who had Recurrence During or Within 6 Months of Completing Adjuvant Oxaliplatin-Based Therapy. Target Oncol (2016) (3):383–400. 10.1007/s11523-015-0402-9 26706237

[9] Grothey A Van Cutsem E Sobrero A Siena S Falcone A Ychou M. Regorafenib monotherapy for previously treated metastatic colorectal cancer (CORRECT): an international, multicentre, randomised, placebo-controlled, phase 3 trial. Lancet (2013) 381(9863):303–12. 10.1016/S0140-6736(12)61900-X 23177514

[10] Tabernero J Yoshino T Cohn AL Obermannova R Bodoky G Garcia-Carbonero R. Ramucirumab versus placebo in combination with second-line FOLFIRI in patients with metastatic colorectal carcinoma that progressed during or after first-line therapy with bevacizumab, oxaliplatin, and a fluoropyrimidine (RAISE): a randomised, double-blind, multicentre, phase 3 study. Lancet Oncol (2015) 16(5):499–508. 10.1016/S1470-2045(15)70127-0 25877855

[11] Cunningham D Humblet Y Siena S Khayat D Bleiberg H Santoro A. Cetuximab monotherapy and cetuximab plus irinotecan in irinotecan-refractory metastatic colorectal cancer. N Engl J Med (2004) 351(4):337–45. 10.1056/NEJMoa033025 15269313

[12] Van Cutsem E Peeters M Siena S Humblet Y Hendlisz A Neyns B. Open-label phase III trial of panitumumab plus best supportive care compared with best supportive care alone in patients with chemotherapy-refractory metastatic colorectal cancer. J Clin Oncol (2007) 25(13):1658–64. 10.1200/JCO.2006.08.1620 17470858

[13] Peters GJ van der Wilt CL van Groeningen CJ Smid K Meijer S Pinedo HM. Thymidylate synthase inhibition after administration of fluorouracil with or without leucovorin in colon cancer patients: implications for treatment with fluorouracil. J Clin Oncol (1994) 12(10):2035–42. 10.1200/JCO.1994.12.10.2035 7931471

[14] Silverstein RA González de Valdivia E Visa N. The Incorporation of 5-Fluorouracil into RNA Affects the Ribonucleolytic Activity of the Exosome Subunit Rrp6. Mol Cancer Res (2011) 9(3):332–40. 10.1158/1541-7786.MCR-10-0084 21289297

[15] Wohlhueter RM McIvor RS Plagemann PG. Facilitated transport of uracil and 5-fluorouracil, and permeation of orotic acid into cultured mammalian cells. J Cell Physiol (1980) 4(3):309–19. 10.1002/jcp.1041040305 7419607

[16] Longley DB Harkin DP Johnston PG. 5-Fluorouracil: mechanisms of action and clinical strategies. Nat Rev Cancer (2003) 3(5):330–8. 10.1038/nrc1074 12724731

[17] Imoto M Azuma H Yamamoto I Otagiri M Imai T. Permeability of 5-fluorouracil and its prodrugs in Caco-2 cell monolayers: evidence for shift from paracellular to transcellular transport by prodrug formation. J Drug Deliv Sci Technol (2009) 19(1):37–41. 10.1016/S1773-2247(09)50005-6

[18] Kerr IG Zimm S Collins JM O’Neill D Poplack DG. Effect of intravenous dose and schedule on cerebrospinal fluid pharmacokinetics of 5-fluorouracil in the monkey. Cancer Res (1984) 44(11):4929–32. 6488155

[19] Xiong H-Y Guo X-L Bu X-X Zhang S-X Ma N-N Song J-R. Autophagic cell death induced by 5-FU in Bax or PUMA deficient human colon cancer cell. Cancer Lett (2010) 288(1):68–74. 10.1016/j.canlet.2009.06.039 19660860

[20] Tang J-C Feng Y-L Liang X Cai X-J. Autophagy in 5-fluorouracil therapy in gastrointestinal cancer: Trends and challenges. Chin Med J (2016) 129(4):456. 10.4103/0366-6999.176069 26879020 PMC4800847

[21] Yang C Pan Y. Fluorouracil induces autophagy-related gastric carcinoma cell death through Beclin-1 upregulation by miR-30 suppression. Tumor Biol (2016) 37(12):15489–94. 10.1007/s13277-015-3775-6 26209295

[22] Cottone L Capobianco A Gualteroni C Perrotta C Bianchi ME Rovere-Querini P. 5-Fluorouracil causes leukocytes attraction in the peritoneal cavity by activating autophagy and HMGB1 release in colon carcinoma cells. Int J Cancer (2015) 136(6):1381–9. 10.1002/ijc.29125 25098891

[23] Nyhan MJ O’Donovan TR Boersma AWM Wiemer EAC McKenna SL. MiR-193b promotes autophagy and non-apoptotic cell death in oesophageal cancer cells. BMC Cancer (2016) 16(1):101. 10.1186/s12885-016-2123-6 26878873 PMC4754993

[24] Focaccetti C Bruno A Magnani E Bartolini D Principi E Dallaglio K. Effects of 5-fluorouracil on morphology, cell cycle, proliferation, apoptosis, autophagy and ROS production in endothelial cells and cardiomyocytes. PloS One (2015) 10(2):e0115686–e. 10.1371/journal.pone.0115686 PMC4324934 25671635

[25] Vodenkova S Buchler T Cervena K Veskrnova V Vodicka P Vymetalkova V. 5-fluorouracil and other fluoropyrimidines in colorectal cancer: Past, present and future. Pharmacol Ther (2020) 206:107447. 10.1016/j.pharmthera.2019.107447 31756363

[26] Sun Z Zhou N Han Q Zhao L Bai C Chen Y. MicroRNA-197 influences 5-fluorouracil resistance via thymidylate synthase in colorectal cancer. Clin Transl Oncol (2015) 17(11):876–83. 10.1007/s12094-015-1318-7 26055341

[27] Zhang W Press OA Haiman CA Yang DY Gordon MA Fazzone W. Association of methylenetetrahydrofolate reductase gene polymorphisms and sex-specific survival in patients with metastatic colon cancer. J Clin Oncol (2007) 25(24):3726–31. 10.1200/JCO.2007.11.4710 17704422

[28] Scartozzi M Maccaroni E Giampieri R Pistelli M Bittoni A Del Prete M. 5-Fluorouracil pharmacogenomics: still rocking after all these years? Pharmacogenomics (2011) 12(2):251–65. 10.2217/pgs.10.167 21332317

[29] Tanaka F Fukuse T Wada H Fukushima M. The history, mechanism and clinical use of oral 5-fluorouracil derivative chemotherapeutic agents. Curr Pharm Biotechnol (2000) 1(2):137–64. 10.2174/1389201003378979 11467334

[30] Santi DV McHenry CS. 5-Fluoro-2′-deoxyuridylate: covalent complex with thymidylate synthetase. Proc Natl Acad Sci (1972) 69(7):1855–7. 10.1073/pnas.69.7.1855 PMC426818 4505665

[31] Milano G McLeod H. Can dihydropyrimidine dehydrogenase impact 5-fluorouracil-based treatment? Eur J Cancer (2000) 36(1):37–42. 10.1016/S0959-8049(99)00211-7 10741292

[32] D RB H BE. Clinical pharmacology of 5-fluorouracil. Clin Pharmacokinet (1989) 16(4):215–37. 10.2165/00003088-198916040-00002 2656050

[33] Fraile RJ Baker LH Buroker TR Horwitz J Vaitkevicius V. Pharmacokinetics of 5-fluorouracil administered orally, by rapid intravenous and by slow infusion. Cancer Res (1980) 40(7):2223–8. 7388790

[34] Wei L Wang X Lv L Liu J Xing H Song Y. The emerging role of microRNAs and long noncoding RNAs in drug resistance of hepatocellular carcinoma. Mol Cancer (2019) 18(1):147. 10.1186/s12943-019-1086-z 31651347 PMC6814027

[35] Yin Q Feng W Shen X Ju S. Regulatory effects of lncRNAs and miRNAs on autophagy in malignant tumorigenesis. Biosci Rep (2018) 38(5):BSR20180516. 10.1042/BSR20180516 30266744 PMC6200703

[36] Thapar R. Regulation of DNA Double-Strand Break Repair by Non-Coding RNAs. Molecules (2018) 23(11):2789. 10.3390/molecules23112789 PMC6278438 30373256

[37] Zhao C Zhao Q Zhang C Wang G Yao Y Huang X. miR-15b-5p resensitizes colon cancer cells to 5-fluorouracil by promoting apoptosis via the NF-κB/XIAP axis. Sci Rep (2017) 7(1):1–12. 10.1038/s41598-017-04172-z 28646148 PMC5482850

[38] Sun L-H Tian D Yang Z-C Li J-L. Exosomal miR-21 promotes proliferation, invasion and therapy resistance of colon adenocarcinoma cells through its target PDCD4. Sci Rep (2020) 10(1):1–8. 10.1038/s41598-020-65207-6 32427870 PMC7237414

[39] Deng J Lei W Fu J-C Zhang L Li J-H Xiong J-P. Targeting miR-21 enhances the sensitivity of human colon cancer HT-29 cells to chemoradiotherapy in vitro. Biochem Biophys Res Commun (2014) 443(3):789–95. 10.1016/j.bbrc.2013.11.064 24275137

[40] Zhao J Cao J Zhou L Du Y Zhang X Yang B. MiR-1260b inhibitor enhances the chemosensitivity of colorectal cancer cells to fluorouracil by targeting PDCD4/IGF1. Oncol Lett (2018) (4):5131–9. 10.3892/ol.2018.9307 PMC6144919 30250581

[41] Zhang H Tang J Li C Kong J Wang J Wu Y. MiR-22 regulates 5-FU sensitivity by inhibiting autophagy and promoting apoptosis in colorectal cancer cells. Cancer Lett (2015) 356(2):781–90. 10.1016/j.canlet.2014.10.029 25449431

[42] Shang J Yang F Wang Y Wang Y Xue G Mei Q. MicroRNA-23a antisense enhances 5-fluorouracil chemosensitivity through APAF-1/caspase-9 apoptotic pathway in colorectal cancer cells. J Cell Biochem (2014) 115(4):772–84. 10.1002/jcb.24721 24249161

[43] Cristóbal I Rubio J Santos A Torrejón B Caramés C Imedio L. MicroRNA-199b Downregulation Confers Resistance to 5-Fluorouracil Treatment and Predicts Poor Outcome and Response to Neoadjuvant Chemoradiotherapy in Locally Advanced Rectal Cancer Patients. Cancers (Basel) (2020) 12(6):1655. 10.3390/cancers12061655 PMC7352382 32580513

[44] Valeri N Gasparini P Braconi C Paone A Lovat F Fabbri M. MicroRNA-21 induces resistance to 5-fluorouracil by down-regulating human DNA MutS homolog 2 (hMSH2). Proc Natl Acad Sci (2010) 107(49):21098–103. 10.1073/pnas.1015541107 PMC3000294 21078976

[45] Zhang Q Li W Liu G Tang W. MicroRNA-24 regulates the growth and chemosensitivity of the human colorectal cancer cells by targeting RNA-binding protein DND1. J Buon (2019) 24:1476–81. 31646794

[46] Wang B Lu F-Y Shi R-H Feng Y-D Zhao X-D Lu Z-P. MiR-26b regulates 5-FU-resistance in human colorectal cancer via down-regulation of Pgp. Am J Cancer Res (2018) 8(12):2518. 30662808 PMC6325481

[47] Bai R Yang Q Xi R Li L Shi D Chen K. miR-941 as a promising biomarker for acute coronary syndrome. BMC Cardiovasc Disord (2017) 17(1):227. 10.1186/s12872-017-0653-8 28830367 PMC5568367

[48] Jiang S Miao D Wang M Lv J Wang Y Tong J. MiR-30-5p suppresses cell chemoresistance and stemness in colorectal cancer through USP 22/Wnt/β-catenin signaling axis. J Cell Mol Med (2019) 23(1):630–40. 10.1111/jcmm.13968 PMC6307779 30338942

[49] Nakagawa Y Kuranaga Y Tahara T Yamashita H Shibata T Nagasaka M. Induced miR-31 by 5-fluorouracil exposure contributes to the resistance in colorectal tumors. Cancer Sci (2019) 110(8):2540. 10.1111/cas.14090 31162779 PMC6676105

[50] Akao Y Noguchi S Iio A Kojima K Takagi T Naoe T. Dysregulation of microRNA-34a expression causes drug-resistance to 5-FU in human colon cancer DLD-1 cells. Cancer Lett (2011) 300(2):197–204. 10.1016/j.canlet.2010.10.006 21067862

[51] Xie Z-Y Wang F-F Xiao Z-H Liu S-F Tang S-L Lai Y-L. Overexpressing microRNA-34a overcomes ABCG2-mediated drug resistance to 5-FU in side population cells from colon cancer via suppressing DLL1. J Biochem (2020) 167(6):557–64. 10.1093/jb/mvaa012 32044957

[52] He J Xie G Tong J Peng Y Huang H Li J. Overexpression of microRNA-122 re-sensitizes 5-FU-resistant colon cancer cells to 5-FU through the inhibition of PKM2 in vitro and in vivo. Cell Biochem Biophys (2014) 70(2):1343–50. 10.1007/s12013-014-0062-x 24898807

[53] Karaayvaz M Zhai H Ju J. miR-129 promotes apoptosis and enhances chemosensitivity to 5-fluorouracil in colorectal cancer. Cell Death Dis (2013) 4(6):e659–e. 10.1038/cddis.2013.193 PMC3702282 23744359

[54] Lv L Li Q Chen S Zhang X Tao X Tang X. miR-133b suppresses colorectal cancer cell stemness and chemoresistance by targeting methyltransferase DOT1L. Exp Cell Res (2019) 385(1):111597. 10.1016/j.yexcr.2019.111597 31525340

[55] Liu B Liu Y Zhao L Pan Y Shan Y Li Y. Upregulation of microRNA-135b and microRNA-182 promotes chemoresistance of colorectal cancer by targeting ST6GALNAC2 via PI3K/AKT pathway. Mol Carcinogen (2017) 56(12):2669–80. 10.1002/mc.22710 28767179

[56] Darabi F Aghaei M Movahedian A Pourmoghadas A Sarrafzadegan N. The role of serum levels of microRNA-21 and matrix metalloproteinase-9 in patients with acute coronary syndrome. Mol Cell Biochem (2016) 422(1-2):51–60. 10.1007/s11010-016-2805-z 27590242

[57] Liu H Yin Y Hu Y Feng Y Bian Z Yao S. miR-139-5p sensitizes colorectal cancer cells to 5-fluorouracil by targeting NOTCH-1. Pathol-Res Pract (2016) 212(7):643–9. 10.1016/j.prp.2016.04.011 27173050

[58] Borralho PM Kren BT Castro RE Moreira da Silva IB Steer CJ Rodrigues CM. MicroRNA-143 reduces viability and increases sensitivity to 5-fluorouracil in HCT116 human colorectal cancer cells. FEBS J (2009) 276(22):6689–700. 10.1111/j.1742-4658.2009.07383.x 19843160

[59] Liu R-L Dong Y Deng Y-Z Wang W-J Li W-D. Tumor suppressor miR-145 reverses drug resistance by directly targeting DNA damage-related gene RAD18 in colorectal cancer. Tumor Biol (2015) 36(7):5011–9. 10.1007/s13277-015-3152-5 25913620

[60] Liu X Xie T Mao X Xue L Chu X Chen L. MicroRNA-149 increases the sensitivity of colorectal cancer cells to 5-fluorouracil by targeting forkhead box transcription factor FOXM1. Cell Physiol Biochem (2016) 39(2):617–29. 10.1159/000445653 27415661

[61] Zhou C Kong W Ju T Xie Q Zhai L. MiR-185-3p mimic promotes the chemosensitivity of CRC cells via AQP5. Cancer Biol Ther (2020) 21(9):790–8. 10.1080/15384047.2020.1761238 PMC7515541 32588739

[62] Kim C Hong Y Lee H Kang H Lee EK. MicroRNA-195 desensitizes HCT116 human colon cancer cells to 5-fluorouracil. Cancer Lett (2018) 412:264–71. 10.1016/j.canlet.2017.10.022 29080751

[63] Feng C Zhang L Sun Y Li X Zhan L Lou Y. GDPD5, a target of miR-195-5p, is associated with metastasis and chemoresistance in colorectal cancer. Biomed Pharmacother (2018) 101:945–52. 10.1016/j.biopha.2018.03.028 29635904

[64] Heydari K Saidijam M reza Sharifi M Asl SS Shabab N Najafi R. The effect of miR-200c inhibition on chemosensitivity (5-fluorouracil) in colorectal cancer. Pathol Oncol Res (2018) 24(1):145–51. 10.1007/s12253-017-0222-6 28411308

[65] Li T Gao F Zhang X-P. miR-203 enhances chemosensitivity to 5-fluorouracil by targeting thymidylate synthase in colorectal cancer. Oncol Rep (2015) 33(2):607–14. 10.3892/or.2014.3646 25482885

[66] Wu H Liang Y Shen L Shen L. MicroRNA-204 modulates colorectal cancer cell sensitivity in response to 5-fluorouracil-based treatment by targeting high mobility group protein A2. Biol Open (2016) 5(5):563–70. 10.1242/bio.015008 PMC4874347 27095441

[67] Meng X Fu R. miR-206 regulates 5-FU resistance by targeting Bcl-2 in colon cancer cells. OncoTargets Ther (2018) 11:1757. 10.2147/OTT.S159093 PMC5881530 29636622

[68] Pranzini E Leo A Rapizzi E Ramazzotti M Magherini F Giovannelli L. miR-210-3p mediates metabolic adaptation and sustains DNA damage repair of resistant colon cancer cells to treatment with 5-fluorouracil. Mol Carcinogen (2019) 58(12):2181–92. 10.1002/mc.23107 31468617

[69] Shen X Zhong J Yu P Zhao Q Huang T. YY1-regulated LINC00152 promotes triple negative breast cancer progression by affecting on stability of PTEN protein. Biochem Biophys Res Commun (2019) 509(2):448–54. 10.1016/j.bbrc.2018.12.074 30594392

[70] Li X Qiu S Zhang X. Overexpression of miR-215-3p sensitizes colorectal cancer to 5-fluorouracil induced apoptosis through regulating CXCR1. Eur Rev Med Pharmacol Sci (2018) 22(21):7240–50. 10.26355/eurrev_201811_16258 30468467

[71] Liu N Li J Zhao Z Han J Jiang T Chen Y. MicroRNA-302a enhances 5-fluorouracil-induced cell death in human colon cancer cells. Oncol Rep (2017) 37(1):631–9. 10.3892/or.2016.5237 27840990

[72] Yin J Shen X Li M Ni F Xu L Lu H. miR-329 regulates the sensitivity of 5-FU in chemotherapy of colorectal cancer by targeting E2F1. In: Oncology letters. Greece: Spandidos Publications (2018). p. 3587–92. 10.3892/ol.2018.9121 PMC6096256 30127965

[73] Xu W Jiang H Zhang F Gao J Hou J. MicroRNA-330 inhibited cell proliferation and enhanced chemosensitivity to 5-fluorouracil in colorectal cancer by directly targeting thymidylate synthase. Oncol Lett (2017) 13(5):3387–94. 10.3892/ol.2017.5895 PMC5431319 28521444

[74] Zhang L Li B Zhang B Zhang H Suo J. miR−361 enhances sensitivity to 5−fluorouracil by targeting the FOXM1−ABCC5/10 signaling pathway in colorectal cancer. Oncol Lett (2019) 18(4):4064–73. 10.3892/ol.2019.10741 PMC6757261 31579069

[75] Xu F Ye ML Zhang YP Li WJ Li MT Wang HZ. MicroRNA-375-3p enhances chemosensitivity to 5-fluorouracil by targeting thymidylate synthase in colorectal cancer. Cancer Sci (2020) 111(5):1528. 10.1111/cas.14356 32073706 PMC7226198

[76] Zhang Y Hu X Miao X Zhu K Cui S Meng Q. Micro RNA-425-5p regulates chemoresistance in colorectal cancer cells via regulation of Programmed Cell Death 10. J Cell Mol Med (2016) 20(2):360–9. 10.1111/jcmm.12742 PMC4727563 26647742

[77] Deng X Li D Ke X Wang Q Yan S Xue Y. Mir-488 alleviates chemoresistance and glycolysis of colorectal cancer by targeting PFKFB3. J Clin Lab Anal (2020) 35(1):e23578. 10.1002/jcla.23578 32990355 PMC7843269

[78] Chai J Dong W Xie C Wang L Han DL Wang S. Micro RNA-494 sensitizes colon cancer cells to fluorouracil through regulation of DPYD. IUBMB Life (2015) 67(3):191–201. 10.1002/iub.1361 25873402

[79] Huang R Lin J Chi Y. miR-519d reduces the 5-fluorouracil resistance in colorectal cancer cells by down-regulating the expression of CCND1. Eur Rev Med Pharmacol Sci (2018) 22(9):2869–75. 10.26355/eurrev_201805_14989 29771440

[80] Liu G Zhou J Dong M. Down-regulation of miR-543 expression increases the sensitivity of colorectal cancer cells to 5-Fluorouracil through the PTEN/PI3K/AKT pathway. Biosci Rep (2019) 39(3):BSR20190249. 10.1042/BSR20190249 30842340 PMC6430726

[81] Zhao P Ma Y-G Zhao Y Liu D Dai Z-J Yan C-Y. MicroRNA-552 deficiency mediates 5-fluorouracil resistance by targeting SMAD2 signaling in DNA-mismatch-repair-deficient colorectal cancer. Cancer Chemother Pharmacol (2019) 84(2):427–39. 10.1007/s00280-019-03866-7 31087138

[82] Jiang H Ju H Zhang L Lu H Jie K. microRNA-577 suppresses tumor growth and enhances chemosensitivity in colorectal cancer. J Biochem Mol Toxicol (2017) 31(6):e21888. 10.1002/jbt.21888 28150434

[83] Zhang Y Talmon G Wang J. MicroRNA-587 antagonizes 5-FU-induced apoptosis and confers drug resistance by regulating PPP2R1B expression in colorectal cancer. Cell Death Dis (2015) 6(8):e1845–e. 10.1038/cddis.2015.200 PMC4558495 26247730

[84] Han J Liu Z Wang N Pan W. MicroRNA-874 inhibits growth, induces apoptosis and reverses chemoresistance in colorectal cancer by targeting X-linked inhibitor of apoptosis protein. Oncol Rep (2016) 36(1):542–50. 10.3892/or.2016.4810 27221209

[85] Zhao J Cao J Zhou L Du Y Zhang X Yang B. MiR−1260b inhibitor enhances the chemosensitivity of colorectal cancer cells to fluorouracil by targeting PDCD4/IGF1. Oncol Lett (2018) 16(4):5131–9. 10.3892/ol.2018.9307 PMC6144919 30250581

[86] Yin J Tang HF Xiang Q Yu J Yang XY Hu N. MiR-122 increases sensitivity of drug-resistant BEL-7402/5-FU cells to 5-fluorouracil via down-regulation of bcl-2 family proteins. Die Pharmazie-An Int J Pharm Sci (2011) 66(12):975–81. 10.1691/ph.2011.1548 22312705

[87] Jiang J-X Gao S Pan Y-Z Yu C Sun C-Y. Overexpression of microRNA-125b sensitizes human hepatocellular carcinoma cells to 5-fluorouracil through inhibition of glycolysis by targeting hexokinase II. Mol Med Rep (2014) 10(2):995–1002. 10.3892/mmr.2014.2271 24865963

[88] Sui C-J Xu F Shen W-F Dai B-H Lu J-J Zhang M-F. MicroRNA-147 suppresses human hepatocellular carcinoma proliferation migration and chemosensitivity by inhibiting HOXC6. Am J Cancer Res (2016) 6(12):2787. 28042500 PMC5199754

[89] Yang X Zang J Pan X Yin J Xiang Q Yu J. miR-503 inhibits proliferation making human hepatocellular carcinoma cells susceptible to 5−fluorouracil by targeting EIF4E. Oncol Rep (2017) 37(1):563–70. 10.3892/or.2016.5220 27840964

[90] Ma J Wang T Guo R Yang X Yin J Yu J. MicroRNA−133a and microRNA−326 co−contribute to hepatocellular carcinoma 5−fluorouracil and cisplatin sensitivity by directly targeting B−cell lymphoma−extra large. Mol Med Rep (2015) 12(4):6235–40. 10.3892/mmr.2015.4134 26239225

[91] Shi L Wu L Chen Z Yang J Chen X Yu F. MiR-141 activates Nrf2-dependent antioxidant pathway via down-regulating the expression of Keap1 conferring the resistance of hepatocellular carcinoma cells to 5-fluorouracil. Cell Physiol Biochem (2015) 35(6):2333–48. 10.1159/000374036 25896253

[92] Zheng R-P Ma D-K Li Z Zhang H-F. MiR-145 Regulates the Chemoresistance of Hepatic Carcinoma Cells Against 5-Fluorouracil by Targeting Toll-Like Receptor 4. Cancer Manage Res (2020) 12:6165. 10.2147/CMAR.S257598 PMC7398893 32801865

[93] Ma K He Y Zhang H Fei Q Niu D Wang D. DNA methylation-regulated miR-193a-3p dictates resistance of hepatocellular carcinoma to 5-fluorouracil via repression of SRSF2 expression. J Biol Chem (2012) 287(8):5639–49. 10.1074/jbc.M111.291229 PMC3285337 22117060

[94] Yang X Yin J Yu J Xiang Q Liu Y Tang S. miRNA-195 sensitizes human hepatocellular carcinoma cells to 5-FU by targeting BCL-w. Oncol Rep (2012) 27(1):250–7. 10.3892/or.2011.1472 21947305

[95] Lee H Kim C Kang H Tak H Ahn S Yoon SK. microRNA-200a-3p increases 5-fluorouracil resistance by regulating dual specificity phosphatase 6 expression. Exp Mol Med (2017) 49(5):e327–e. 10.1038/emm.2017.33 PMC5454440 28496200

[96] Cai D He K Se C Tong D Huang C. MicroRNA-302b enhances the sensitivity of hepatocellular carcinoma cell lines to 5-FU via targeting Mcl-1 and DPYD. Int J Mol Sci (2015) 16(10):23668–82. 10.3390/ijms161023668 PMC4632720 26457704

[97] Korourian A Madjd Z Roudi R Shariftabrizi A Soleimani M. Induction of miR-31 causes increased sensitivity to 5-FU and decreased migration and cell invasion in gastric adenocarcinoma. Bratislava Med J (2019) 120(1):35–9. 10.4149/BLL_2019_005 30685990

[98] Shen J Niu W Zhang H Jun M Zhang H. Downregulation of microRNA-147 inhibits cell proliferation and increases the chemosensitivity of gastric cancer cells to 5-fluorouracil by directly targeting PTEN. Oncol Res Featuring Preclin Clin Cancer Ther (2018) 26(6):901–11. 10.3727/096504017X15061902533715 PMC7844761 28950928

[99] Wang X-Y Zhou Y-C Wang Y Liu Y-Y Wang Y-X Chen D-D. miR-149 contributes to resistance of 5-FU in gastric cancer via targeting TREM2 and regulating β-catenin pathway. Biochem Biophys Res Commun (2020) 532(3):329–35. 10.1016/j.bbrc.2020.05.135 32977944

[100] Wang C. MiR-195 reverses 5-FU resistance through targeting HMGA1 in gastric cancer cells. Eur Rev Med Pharmacol Sci (2019) 23(9):3771–8. 10.26355/eurrev_201905_17803 31115003

[101] Yu X Shi W Zhang Y Wang X Sun S Song Z. CXCL12/CXCR4 axis induced miR-125b promotes invasion and confers 5-fluorouracil resistance through enhancing autophagy in colorectal cancer. Sci Rep (2017) 7(1):1–13. 10.1038/srep42226 28176874 PMC5296742

[102] Nie H Mu J Wang J Li Y. miR−195−5p regulates multi−drug resistance of gastric cancer cells via targeting ZNF139. Oncol Rep (2018) 40(3):1370–8. 10.3892/or.2018.6524 PMC6072402 29956811

[103] Xiong HL Zhou SW Sun AH He Y Li J Yuan X. MicroRNA−197 reverses the drug resistance of fluorouracil−induced SGC7901 cells by targeting mitogen−activated protein kinase 1. Mol Med Rep (2015) 12(4):5019–25. 10.3892/mmr.2015.4052 PMC4581796 26151540

[104] Li L-Q Pan D Chen Q Zhang S-W Xie D-Y Zheng X-L. Sensitization of gastric cancer cells to 5-FU by microRNA-204 through targeting the TGFBR2-mediated epithelial to mesenchymal transition. Cell Physiol Biochem (2018) 47(4):1533–45. 10.1159/000490871 29940566

[105] Zhang F Li K Yao X Wang H Li W Wu J. A miR-567-PIK3AP1-PI3K/AKT-c-Myc feedback loop regulates tumour growth and chemoresistance in gastric cancer. EBioMedicine (2019) 44:311–21. 10.1016/j.ebiom.2019.05.003 PMC6603849 31078520

[106] Jiang L Yang W Bian W Yang H Wu X Li Y. microRNA-623 targets cyclin D1 to inhibit cell proliferation and enhance the chemosensitivity of cells to 5-fluorouracil in gastric cancer. Oncol Res Featuring Preclin Clin Cancer Ther (2018) 27(1):19–27. 10.3727/096504018X15193469240508 PMC7848397 29495973

[107] Gong X Xu B Zi L Chen X. miR-625 reverses multidrug resistance in gastric cancer cells by directly targeting ALDH1A1. Cancer Manage Res (2019) 11:6615. 10.2147/CMAR.S208708 PMC6643062 31410057

[108] Nishibeppu K Komatsu S Imamura T Kiuchi J Kishimoto T Arita T. Plasma microRNA profiles: identification of miR-1229-3p as a novel chemoresistant and prognostic biomarker in gastric cancer. Sci Rep (2020) 10(1):1–11. 10.1038/s41598-020-59939-8 32081926 PMC7035283

[109] Li B Hong P Zheng C-C Dai W Chen W-Y Yang Q-S. Identification of miR-29c and its Target FBXO31 as a Key Regulatory Mechanism in Esophageal Cancer Chemoresistance: Functional Validation and Clinical Significance. Theranostics (2019) 9(6):1599–613. 10.7150/thno.30372 PMC6485198 31037126

[110] Golubovskaya VM Sumbler B Ho B Yemma M Cance WG. MiR-138 and MiR-135 directly target focal adhesion kinase, inhibit cell invasion, and increase sensitivity to chemotherapy in cancer cells. Anti-Cancer Agents Med Chem (2014) 14(1):18–28. 10.2174/187152061401140108113435 PMC3883917 23438844

[111] Zhao L Zou D Wei X Wang L Zhang Y Liu S. MiRNA-221-3p desensitizes pancreatic cancer cells to 5-fluorouracil by targeting RB1. Tumor Biol (2016) 37(12):16053–63. 10.1007/s13277-016-5445-8 27726102

[112] Wang W Liu B Sun S Lan L Chen Y Han S. Downregulation of miR-486-5p Enhances the Anti-Tumor Effect of 5-Fluorouracil on Pancreatic Cancer Cells. OncoTargets Ther (2020) 13:1649. 10.2147/OTT.S231153 PMC7047986 32158231

[113] Wei X Wang W Wang L Zhang Y Zhang X Chen M. Micro RNA-21 induces 5-fluorouracil resistance in human pancreatic cancer cells by regulating PTEN and PDCD 4. Cancer Med (2016) 5(4):693–702. 10.1002/cam4.626 26864640 PMC4831288

[114] Wang W Zhao L Wei X Wang L Liu S Yang Y. MicroRNA-320a promotes 5-FU resistance in human pancreatic cancer cells. Sci Rep (2016) 6(1):1–11. 10.1038/srep27641 27279541 PMC4899709

[115] Ma J Wu D Yi J Yi Y Zhu X Qiu H. MiR-378 promoted cell proliferation and inhibited apoptosis by enhanced stem cell properties in chronic myeloid leukemia K562 cells. Biomed Pharmacother (2019) 112:108623. 10.1016/j.biopha.2019.108623 30797151

[116] Chen Q Hou J Wu Z Zhao J Ma D. miR-145 Regulates the sensitivity of esophageal squamous cell carcinoma cells to 5-FU via targeting REV3L. Pathol-Res Pract (2019) 215(7):152427. 10.1016/j.prp.2019.04.019 31072625

[117] Han L Cui D Li B Xu WW Lam AKY Chan KT. MicroRNA-338-5p reverses chemoresistance and inhibits invasion of esophageal squamous cell carcinoma cells by targeting Id-1. Cancer Sci (2019) 110(12):3677. 10.1111/cas.14220 31646712 PMC6890449

[118] Chen B Duan L Yin G Tan J Jiang X. miR-381, a novel intrinsic WEE1 inhibitor, sensitizes renal cancer cells to 5-FU by up-regulation of Cdc2 activities in 786-O. J Chemothe (2013) 25(4):229–38. 10.1179/1973947813Y.0000000092 23816136

[119] Luan W Qian Y Ni X Bu X Xia Y Wang J. miR-204-5p acts as a tumor suppressor by targeting matrix metalloproteinases-9 and B-cell lymphoma-2 in malignant melanoma. OncoTargets Ther (2017) 10:1237. 10.2147/OTT.S128819 PMC5338948 28280358

[120] Wang W Chen L Qian J Zhang Q. MiR-335 promotes cell proliferation by inhibiting MEF2D and sensitizes cells to 5-Fu treatment in gallbladder carcinoma. Eur Rev Med Pharmacol Sci (2019) 23(22):9829–39. 10.26355/eurrev_201911_19546 31799650

[121] Gotanda K Hirota T Matsumoto N Ieiri I. MicroRNA-433 negatively regulates the expression of thymidylate synthase (TYMS) responsible for 5-fluorouracil sensitivity in HeLa cells. BMC Cancer (2013) 13(1):369. 10.1186/1471-2407-13-369 23915286 PMC3750578

[122] Xiong Y Gu Y Wang F Li L Zhu M Wang N. LINC01857 as an oncogene regulates CREB1 activation by interacting with CREBBP in breast cancer. J Cell Physiol (2019) 234(8):14031–9. 10.1002/jcp.28090 PMC13483081 30628071

[123] Ren T Hou J Liu C Shan F Xiong X Qin A. The long non-coding RNA HOTAIRM1 suppresses cell progression via sponging endogenous miR-17-5p/B-cell translocation gene 3 (BTG3) axis in 5-fluorouracil resistant colorectal cancer cells. Biomed Pharmacother (2019) 117:109171. 10.1016/j.biopha.2019.109171 31261026

[124] Li P Zhang X Wang L Du L Yang Y Liu T. lncRNA HOTAIR contributes to 5FU resistance through suppressing miR-218 and activating NF-κB/TS signaling in colorectal cancer. Mol Ther-Nucl Acids (2017) 8:356–69. 10.1016/j.omtn.2017.07.007 PMC5537205 28918035

[125] Xiong W Jiang Y-X Ai Y-Q Liu S Wu X-R Cui J-G. Microarray analysis of long non-coding RNA expression profile associated with 5-fluorouracil-based chemoradiation resistance in colorectal cancer cells. Asian Pac J Cancer Prev (2015) 16(8):3395–402. 10.7314/APJCP.2015.16.8.3395 25921151

[126] Wu H Zou Q He H Liang Y Lei M Zhou Q. Long non-coding RNA PCAT6 targets miR-204 to modulate the chemoresistance of colorectal cancer cells to 5-fluorouracil-based treatment through HMGA2 signaling. Cancer Med (2019) 8(5):2484–95. 10.1002/cam4.1809 PMC6536993 30938104

[127] Liu F Ai FY Zhang DC Tian L Yang ZY Liu SJ. LncRNA NEAT1 knockdown attenuates autophagy to elevate 5-FU sensitivity in colorectal cancer via targeting miR-34a. Cancer Med (2020) 9(3):1079–91. 10.1002/cam4.2746 PMC6997058 31802650

[128] Wang X Jiang G Ren W Wang B Yang C Li M. LncRNA NEAT1 Regulates 5-Fu Sensitivity, Apoptosis and Invasion in Colorectal Cancer Through the MiR-150-5p/CPSF4 Axis. OncoTargets Ther (2020) 13:6373. 10.2147/OTT.S239432 PMC7336013 32669857

[129] Li J Li X Cen C Ai X Lin C Hu G. The long non-coding RNA ENST00000547547 reduces 5-fluorouracil resistance of colorectal cancer cells via competitive binding to microRNA-31. Oncol Rep (2018) 39(1):217–26. 10.3892/or.2017.6082 29115526

[130] Bian Z Jin L Zhang J Yin Y Quan C Hu Y. LncRNA—UCA1 enhances cell proliferation and 5-fluorouracil resistance in colorectal cancer by inhibiting miR-204-5p. Sci Rep (2016) 6:23892. 10.1038/srep23892 27046651 PMC4820696

[131] Xiao Y Yurievich UA Yosypovych SV. Long noncoding RNA XIST is a prognostic factor in colorectal cancer and inhibits 5-fluorouracil-induced cell cytotoxicity through promoting thymidylate synthase expression. Oncotarget (2017) 8(47):83171. 10.18632/oncotarget.20487 29137332 PMC5669958

[132] Wang M Hu H Wang Y Huang Q Huang R Chen Y. Long non-coding RNA TUG1 mediates 5-fluorouracil resistance by acting as a ceRNA of miR-197-3p in colorectal cancer. J Cancer (2019) 10(19):4603. 10.7150/jca.32065 31528224 PMC6746119

[133] Jiang Z Li L Hou Z Liu W Wang H Zhou T. LncRNA HAND2-AS1 inhibits 5-fluorouracil resistance by modulating miR-20a/PDCD4 axis in colorectal cancer. Cell Signall (2020) 66:109483. 10.1016/j.cellsig.2019.109483 31760170

[134] Bian Z Zhang J Li M Feng Y Yao S Song M. Long non-coding RNA LINC00152 promotes cell proliferation, metastasis, and confers 5-FU resistance in colorectal cancer by inhibiting miR-139-5p. Oncogenesis (2017) 6(11):1–11. 10.1038/s41389-017-0008-4 PMC5868057 29180678

[135] Wang M Han D Yuan Z Hu H Zhao Z Yang R. Long non-coding RNA H19 confers 5-Fu resistance in colorectal cancer by promoting SIRT1-mediated autophagy. Cell Death Dis (2018) 9(12):1–14. 10.1038/s41419-018-1187-4 30451820 PMC6242979

[136] Yang C Pan Y Deng SP. Downregulation of lncRNA CCAT1 enhances 5-fluorouracil sensitivity in human colon cancer cells. BMC Mol Cell Biol (2019) 20(1):1–11. 10.1186/s12860-019-0188-1 31039730 PMC6480879

[137] Yokoyama Y Sakatani T Wada R Ishino K Kudo M Koizumi M. In vitro and in vivo studies on the association of long non−coding RNAs H19 and urothelial cancer associated 1 with the susceptibility to 5−fluorouracil in rectal cancer. Int J Oncol (2019) 55(6):1361–71. 10.3892/ijo.2019.4895 31638183

[138] Yu J Shen J Qiao X Cao L Yang Z Ye H. SNHG20/miR−140−5p/NDRG3 axis contributes to 5−fluorouracil resistance in gastric cancer. Oncol Lett (2019) 18(2):1337–43. 10.3892/ol.2019.10439 PMC6607387 31423195

[139] Du P Hu CE Qin Y Zhao J Patel R Fu Y. LncRNA PVT1 mediates antiapoptosis and 5-Fluorouracil resistance via increasing Bcl2 expression in gastric cancer. J Oncol (2019) 2019:9325407. 10.1155/2019/9325407 31205469 PMC6530232

[140] Wu L Pan C Wei X Shi Y Zheng J Lin X. lncRNA KRAL reverses 5-fluorouracil resistance in hepatocellular carcinoma cells by acting as a ceRNA against miR-141. Cell Commun Signaling (2018) 16(1):47. 10.1186/s12964-018-0260-z PMC6098660 30119680

[141] Quan D Chen K Zhang J Guan Y Yang D Wu H. Identification of lncRNA NEAT1/miR-21/RRM2 axis as a novel biomarker in breast cancer. J Cell Physiol (2020) 235(4):3372–81. 10.1002/jcp.29225 PMC13482681 31621912

[142] Li H Zhou Y Cheng H Tian J Yang S. Roles of a TMPO-AS1/microRNA-200c/TMEFF2 ceRNA network in the malignant behaviors and 5-FU resistance of ovarian cancer cells. Exp Mol Pathol (2020) Aug 115:104481. 10.1016/j.yexmp.2020.104481 32497621

[143] Zhao W Dong S Duan B Chen P Shi L Gao H. HOTAIR is a predictive and prognostic biomarker for patients with advanced gastric adenocarcinoma receiving fluorouracil and platinum combination chemotherapy. Am J Trans Res (2015) 7(7):1295. PMC4548321 26328013

[144] Xu Z Liu C Zhao Q Lü J Ding X Luo A. Long non-coding RNA CCAT2 promotes oncogenesis in triple-negative breast cancer by regulating stemness of cancer cells. Pharmacol Res (2020) 152:104628. 10.1016/j.phrs.2020.104628 31904506

[145] Lin K Jiang H Zhuang S-S Qin Y-S Qiu G-D She Y-Q. Long noncoding RNA LINC00261 induces chemosensitization to 5-fluorouracil by mediating methylation-dependent repression of DPYD in human esophageal cancer. FASEB J (2019) 33(2):1972–88. 10.1096/fj.201800759R 30226808

[146] Zhang S Zheng F Zhang L Huang Z Huang X Pan Z. LncRNA HOTAIR-mediated MTHFR methylation inhibits 5-fluorouracil sensitivity in esophageal cancer cells. J Exp Clin Cancer Res (2020) 39(1):1–13. 10.1186/s13046-020-01610-1 32653028 PMC7353690

[147] Chen J-L Lin Z-X Qin Y-S She Y-Q Chen Y Chen C. Overexpression of long noncoding RNA LINC01419 in esophageal squamous cell carcinoma and its relation to the sensitivity to 5-fluorouracil by mediating GSTP1 methylation. Ther Adv Med Oncol (2019) 11:1758835919838958. 10.1177/1758835919838958 31019568 PMC6463338

[148] Yong S Yabin Y Bing Z Chuanrong Z Dianhua G Jianhuai Z. Reciprocal regulation of DGCR5 and miR-320a affects the cellular malignant phenotype and 5-FU response in pancreatic ductal adenocarcinoma. Oncotarget (2017) 8(53):90868. 10.18632/oncotarget.18377 29207609 PMC5710890

[149] Zhang J Zhou W Wang X Wang L. The CRISPR-Cas9 system: a promising tool for discovering potential approaches to overcome drug resistance in cancer. RSC Adv (2018) 8(58):33464–72. 10.1039/C8RA04509G PMC9086466 35548117

[150] Chen Y Zhang Y. Application of the CRISPR/Cas9 system to drug resistance in breast cancer. Adv Sci (2018) 5(6):1700964. 10.1002/advs.201700964 PMC6010891 29938175

[151] Saber A Liu B Ebrahimi P Haisma HJ. CRISPR/Cas9 for overcoming drug resistance in solid tumors. DARU J Pharm Sci (2020) 28(1):295–304. 10.1007/s40199-019-00240-z PMC7214581 30666557

[152] Gaj T Gersbach CA Barbas CF. ZFN II. TALEN, and CRISPR/Cas-based methods for genome engineering. Trends Biotechnol (2013) 31(7):397–405. 10.1016/j.tibtech.2013.04.004 23664777 PMC3694601

[153] Nemudryi A Valetdinova K Medvedev S Zakian S. TALEN and CRISPR/Cas genome editing systems: tools of discovery. Acta Naturae (2014) 6(3):22. 10.32607/20758251-2014-6-3-19-40 PMC4207558 25349712

[154] Goulin EH Galdeano DM Granato LM Matsumura EE Dalio RJD Machado MA. RNA interference and CRISPR: Promising approaches to better understand and control citrus pathogens. Microbiol Res (2019) 226:1–9. 10.1016/j.micres.2019.03.006 31284938

[155] Liu T-T Liu X-S Zhang M Liu X-N Zhu F-X Zhu F-M. Cartilage oligomeric matrix protein is a prognostic factor and biomarker of colon cancer and promotes cell proliferation by activating the Akt pathway. J Cancer Res Clin Oncol (2018) 144(6):1049–63. 10.1007/s00432-018-2626-4 PMC11813401 29560517

[156] Mu H He Y Wang S Yang S Wang Y Nan C. MiR-130b/TNF-α/NF-κB/VEGFA loop inhibits prostate cancer angiogenesis. Clin Trans Oncol (2020) 22(1):111–21. 10.1007/s12094-019-02217-5 31667686

[157] Liu B Yang H Pilarsky C Weber GF. The effect of GPRC5a on the proliferation, migration ability, chemotherapy resistance, and phosphorylation of GSK-3β in pancreatic Cancer. Int J Mol Sci (2018) 19(7):1870. 10.3390/ijms19071870 PMC6073545 29949874

[158] Pothuraju R Rachagani S Krishn SR Chaudhary S Nimmakayala RK Siddiqui JA. Molecular implications of MUC5AC-CD44 axis in colorectal cancer progression and chemoresistance. Mol Cancer (2020) 19(1):1–14. 10.1186/s12943-020-01156-y 32098629 PMC7041280

[159] Saeinasab M Bahrami AR González J Marchese FP Martinez D Mowla SJ. SNHG15 is a bifunctional MYC-regulated noncoding locus encoding a lncRNA that promotes cell proliferation, invasion and drug resistance in colorectal cancer by interacting with AIF. J Exp Clin Cancer Res (2019) 38(1):1–16. 10.1186/s13046-019-1169-0 31014355 PMC6480895

[160] Satapathy SR Sjölander A. Cysteinyl leukotriene receptor 1 promotes 5-fluorouracil resistance and resistance-derived stemness in colon cancer cells. Cancer Lett (2020) 28(448):50–62. 10.1016/j.canlet.2020.05.023 32474153

[161] Lai Y Chen Y Lin Y Ye L. Down-regulation of LncRNA CCAT1 enhances radiosensitivity via regulating miR-148b in breast cancer. Cell Biol Int (2018) 42(2):227–36. 10.1002/cbin.10890 29024383

[162] Kaller M Götz U Hermeking H. Loss of p53-inducible long non-coding RNA LINC01021 increases chemosensitivity. Oncotarget (2017) 8(61):102783. 10.18632/oncotarget.22245 29262524 PMC5732690

[163] Celestini V Tezil T Russo L Fasano C Sanese P Forte G. Uncoupling FoxO3A mitochondrial and nuclear functions in cancer cells undergoing metabolic stress and chemotherapy. Cell Death Dis (2018) 9(2):1–20. 10.1038/s41419-018-0336-0 29445193 PMC5833443

[164] Lobo S Pereira C Oliveira C Almeida GM. Skipping Exon-v6 from CD44v6-Containing Isoforms Influences Chemotherapy Response and Self-Renewal Capacity of Gastric Cancer Cells. Cancers (Basel) (2020) 12(9):2378. 10.3390/cancers12092378 PMC7564355 32842638

[165] Wang Y Yin B Li D Wang G Han X Sun X. GSDME mediates caspase-3-dependent pyroptosis in gastric cancer. Biochem Biophys Res Commun (2018) 495(1):1418–25. 10.1016/j.bbrc.2017.11.156 29183726

[166] Miao Z-F Sun J-X Adkins-Threats M Pang M-J Zhao J-H Wang X. DDIT4 licenses only healthy cells to proliferate during injury-induced metaplasia. Gastroenterology (2020) 160(1):260–71. 10.1053/j.gastro.2020.09.016 PMC7857017 32956680

[167] Wu Z-J Zhao X Banaszak LG Gutierrez-Rodrigues F Keyvanfar K Gao S-G. CRISPR/Cas9-mediated ASXL1 mutations in U937 cells disrupt myeloid differentiation. Int J Oncol (2018) 52(4):1209–23. 10.3892/ijo.2018.4290 PMC5843401 29532865

[168] Yuen K-S Wang Z-M Wong N-HM Zhang Z-Q Cheng T-F Lui W-Y. Suppression of Epstein-Barr virus DNA load in latently infected nasopharyngeal carcinoma cells by CRISPR/Cas9. Virus Res (2018) 244:296–303. 10.1016/j.virusres.2017.04.019 28456574

[169] Liu DS Read M Cullinane C Azar WJ Fennell CM Montgomery KG. APR-246 potently inhibits tumour growth and overcomes chemoresistance in preclinical models of oesophageal adenocarcinoma. Gut (2015) 64(10):1506–16. 10.1136/gutjnl-2015-309770 26187504

[170] Wang K Xing Z-H Jiang Q-W Yang Y Huang J-R Yuan M-L. Targeting uPAR by CRISPR/Cas9 system attenuates cancer malignancy and multidrug resistance. Front Oncol (2019) 9:80. 10.3389/fonc.2019.00080 30873379 PMC6400983

[171] Eckschlager T Plch J Stiborova M Hrabeta J. Histone deacetylase inhibitors as anticancer drugs. Int J Mol Sci (2017) 18(7):1414. 10.3390/ijms18071414 PMC5535906 28671573

[172] Yoshida M Furumai R Nishiyama M Komatsu Y Nishino N Horinouchi S. Histone deacetylase as a new target for cancer chemotherapy. Cancer Chemother Pharmacol (2001) 48(1):S20–S6. 10.1007/s002800100300 11587361

[173] Ocker M Alajati A Ganslmayer M Zopf S Lüders M Neureiter D. The histone-deacetylase inhibitor SAHA potentiates proapoptotic effects of 5-fluorouracil and irinotecan in hepatoma cells. J Cancer Res Clin Oncol (2005) 131(6):385–94. 10.1007/s00432-004-0664-6 PMC12161252 15754201

[174] Minegaki T Suzuki A Mori M Tsuji S Yamamoto S Watanabe A. Histone deacetylase inhibitors sensitize 5−fluorouracil−resistant MDA−MB−468 breast cancer cells to 5−fluorouracil. Oncol Lett (2018) 16(5):6202–8. 10.3892/ol.2018.9388 PMC6176421 30333885

[175] Okada K Hakata S Terashima J Gamou T Habano W Ozawa S. Combination of the histone deacetylase inhibitor depsipeptide and 5-fluorouracil upregulates major histocompatibility complex class II and p21 genes and activates caspase-3/7 in human colon cancer HCT-116 cells. Oncol Rep (2016) 36(4):1875–85. 10.3892/or.2016.5008 PMC5022900 27509880

[176] Hamam R Ali D Vishnubalaji R Alsaaran ZF Chalisserry EP Alfayez M. Enhanced efficacy of 5-fluorouracil in combination with a dual histone deacetylase and phosphatidylinositide 3-kinase inhibitor (CUDC-907) in colorectal cancer cells. Saudi J Gastroenterol (2017) 23(1):34. 10.4103/1319-3767.199136 28139498 PMC5329975

[177] Tan Y Zhang S Zhu H Chu Y Zhou H Liu D. Histone deacetylase 6 selective inhibitor ACY1215 inhibits cell proliferation and enhances the chemotherapeutic effect of 5-fluorouracil in HCT116 cells. Ann Transl Med (2019) 7(1):2. 10.21037/atm.2018.11.48 30788349 PMC6351378

[178] Yu S-L Lee DC Son JW Park CG Lee HY Kang J. Histone deacetylase 4 mediates SMAD family member 4 deacetylation and induces 5-fluorouracil resistance in breast cancer cells. Oncol Rep (2013) 30(3):1293–300. 10.3892/or.2013.2578 23817620

[179] Li Y-J Lei Y-H Yao N Wang C-R Hu N Ye W-C. Autophagy and multidrug resistance in cancer. Chin J Cancer (2017) 36(1):52. 10.1186/s40880-017-0219-2 28646911 PMC5482965

[180] Panzarini E Dini L. Nanomaterial-induced autophagy: a new reversal MDR tool in cancer therapy? Mol Pharm (2014) 11(8):2527–38. 10.1021/mp500066v 24921216

[181] Kumar P Zhang D-M Degenhardt K Chen Z-S. Autophagy and transporter-based multi-drug resistance. Cells (2012) 1(3):558–75. 10.3390/cells1030558 PMC3901113 24710490

[182] Yang X Sun Y Zhang Y Han S. Downregulation of miR−181b inhibits human colon cancer cell proliferation by targeting CYLD and inhibiting the NF−κB signaling pathway. Int J Mol Med (2020) 46(5):1755–64. 10.3892/ijmm.2020.4720 PMC7521473 32901872

[183] Jin C Wang A Liu L Wang G Li G Han Z. miR-145-5p inhibits tumor occurrence and metastasis through the NF-κB signaling pathway by targeting TLR4 in malignant melanoma. J Cell Biochem (2019) 120(7):11115–26. 10.1002/jcb.28388 30701576

[184] Zhang P Lai Z-L Chen H-F Zhang M Wang A Jia T. Curcumin synergizes with 5-fluorouracil by impairing AMPK/ULK1-dependent autophagy, AKT activity and enhancing apoptosis in colon cancer cells with tumor growth inhibition in xenograft mice. J Exp Clin Cancer Res (2017) 36(1):190. 10.1186/s13046-017-0661-7 29273065 PMC5741949

[185] Kim AY Kwak J-H Je NK Lee Y-H Jung Y-S. Epithelial-mesenchymal Transition is Associated with Acquired Resistance to 5-Fluorocuracil in HT-29 Colon Cancer Cells. Toxicol Res (2015) 31(2):151–6. 10.5487/TR.2015.31.2.151 PMC4505345 26191381

[186] Liang G Zhu Y Ali DJ Tian T Xu H Si K. Engineered exosomes for targeted co-delivery of miR-21 inhibitor and chemotherapeutics to reverse drug resistance in colon cancer. J Nanobiotechnol (2020) 18(1):1–15. 10.1186/s12951-019-0563-2 PMC6950820 31918721

[187] Fettke H Kwan EM Azad AA. Cell-free DNA in cancer: current insights. Cell Oncol (2019) 42(1):13–28. 10.1007/s13402-018-0413-5 PMC12994338 30367445

[188] Yousefi M Ghaffari P Nosrati R Dehghani S Salmaninejad A Abarghan YJ. Prognostic and therapeutic significance of circulating tumor cells in patients with lung cancer. Cell Oncol (2020) 43(1):31–9. 10.1007/s13402-019-00470-y PMC12990721 31828552

